# *Yarrowia lipolytica* Strains and Their Biotechnological Applications: How Natural Biodiversity and Metabolic Engineering Could Contribute to Cell Factories Improvement

**DOI:** 10.3390/jof7070548

**Published:** 2021-07-10

**Authors:** Catherine Madzak

**Affiliations:** Université Paris-Saclay, INRAE, AgroParisTech, UMR SayFood, F-78850 Thiverval-Grignon, France; catherine.madzak@inrae.fr

**Keywords:** white biotechnology, metabolic engineering, non-conventional yeast, oleaginous yeast, cell factory, heterologous expression, biodiversity, *Yarrowia lipolytica*, *Yarrowia* clade, GMO

## Abstract

Among non-conventional yeasts of industrial interest, the dimorphic oleaginous yeast *Yarrowia lipolytica* appears as one of the most attractive for a large range of white biotechnology applications, from heterologous proteins secretion to cell factories process development. The past, present and potential applications of wild-type, traditionally improved or genetically modified *Yarrowia lipolytica* strains will be resumed, together with the wide array of molecular tools now available to genetically engineer and metabolically remodel this yeast. The present review will also provide a detailed description of *Yarrowia lipolytica* strains and highlight the natural biodiversity of this yeast, a subject little touched upon in most previous reviews. This work intends to fill this gap by retracing the genealogy of the main *Yarrowia lipolytica* strains of industrial interest, by illustrating the search for new genetic backgrounds and by providing data about the main publicly available strains in yeast collections worldwide. At last, it will focus on exemplifying how advances in engineering tools can leverage a better biotechnological exploitation of the natural biodiversity of *Yarrowia lipolytica* and of other yeasts from the *Yarrowia* clade.

## 1. Introduction

A major challenge for our societies is to replace polluting technologies, based on fossil fuels, with clean ones, based on renewable resources. White biotechnology, using microorganisms and their enzymes to manufacture compounds of industrial interest (chemicals, biomaterials, biofuels, pharmaceuticals, feed, food), has an important role to play in this transition. This rapidly developing field aims to design industrial processes more environmentally friendly and making use of agricultural, forest and industrial waste or by-products. Among the microorganisms amenable for such industrial applications, yeasts cells present the cumulated advantages of high growth capacity, easy genetic manipulation and presence of a eukaryotic organisation allowing posttranslational processing, vesicular secretion and subcellular compartmentalization.

After having drawn some industrialists’ attention as early as the 1950s, the non-conventional oleaginous yeast *Yarrowia lipolytica* has been recognized since several decades, as a powerful host for heterologous protein expression, secretion and surface display. The development of sequencing and genetic engineering tools, combined with an increasing knowledge of its metabolism, have then facilitated the complex engineering of the metabolic pathways of this yeast for various applications. Since nearly two decades, numerous laboratories throughout the world have chosen *Y. lipolytica* as a chassis for designing microbial cell factories. White biotechnology applications of this yeast include notably single cell oil production, whole cell bioconversion and upgrading of industrial wastes. This history of use will be briefly resumed, but the present publication intends to put a new emphasis on the choice of available *Yarrowia lipolytica* strains and the natural biodiversity of this species. This review will present the various *Y. lipolytica* strains of industrial interest, retrace their genealogy and resume their preferred applications. As the numerous molecular tools available for the genetic engineering of this yeast have already been abundantly described elsewhere, this review will focus on those that leverage a better biotechnological exploitation of the natural biodiversity of this species and, possibly, of other yeasts from the *Yarrowia* clade.

## 2. Everything You Always Wanted to Know about *Yarrowia lipolytica* (Briefly Resumed)

### 2.1. Natural Habitats and Safety

*Y. lipolytica* is a Crabtree-negative ascomycete yeast (class: Saccharomycetes, order: Saccharomycetales) that has been at first noticed for its remarkable lipolytic and proteolytic capacities. In accordance to these high levels of secreted enzymatic activities, wild-type isolates of this yeast generally originate from lipid-rich and/or protein-rich environments, notably from meat and dairy products (especially fermented ones, such as dry sausages and cheeses) and from sewage or oil-polluted waters [1,2]. In the last decades, the range of ecosystems from which *Y. lipolytica* strains has been isolated has broadened to encompass very diverse habitats, from marine waters, salt marshes and soils (especially oil-polluted ones) to a variety of consumable products (including fruits, vegetables or seafood) and even the excreta of insects or vertebrates that consume them [1,3,4,5,6,7]. This species thus appears to exhibit a rather ubiquitous distribution, in the natural world as well as in man-made extreme environments. The ecological significance of *Y. lipolytica* has been reviewed very recently, establishing this yeast as an eco-friendly organism able to develop symbiosis with some insects (beetles) and plants (microbial endophyte, mycorhizes) [7].

Since only a decade, *Y. lipolytica* is also considered as belonging to the normal human mycobiota, being found notably in the mouth and respiratory tract of adults, especially of diabetic people [8]. This yeast is also sometimes seen as a possible opportunistic emerging pathogen, since its biofilm formation capacity can be responsible of rare cases of catheter-related candidaemia [1,8]. Despite this, *Y. lipolytica* is classified as a Biosafety Level (BSL) 1 microorganism by the Public Health Service (Washington, DC, USA). It is also recognized as a “microorganism with a documented use in food” by the International Dairy Federation (IDF) and the European Food and Feed Cultures Association (EFFCA), and as a “recommended biological agent for production purposes” by the European Food Safety Authority. This yeast has also gained a GRAS (generally recognized as safe) status, from the many GRAS notifications for its various applications that have been approved by the USA Food and Drug Administration (FDA) [1,8].

### 2.2. Main Characteristics

#### 2.2.1. Physico-Chemical Conditions for Growth

In contrast with most other hemiascomycetous yeasts, *Y. lipolytica* is an obligate aerobe, for which oxygen concentration constitute a limiting factor for growth. Its temperature limit is in the range of 32 to 34 °C for most strains, although a very few can grow as high as 37 °C. Most strains can be considered as psychrotrophic since they exhibit a residual growth when kept at 4–5 °C. The preferred growth temperature is however in the range of 25 to 30 °C [9]. *Y. lipolytica* is able to grow at a large range of pHs: most strains can be cultivated at pH 3.5 to 8.0 and a few can tolerate lower ones (2.0) or even very high pHs (9.7) [9]. In accordance with its presence in salty environments and foods, *Y. lipolytica* tolerates high salt concentrations, such as 7.5% NaCl for most strains and as high as 15% NaCl for a few of them [9]. This yeast is also known to be able to adsorb metallic atoms and has therefore been proposed for bioremediation of wastes containing heavy metals such as Cr, Fe, Ni, Cu, Zn and Cd [9].

#### 2.2.2. Ploidy and Morphology

This yeast is heterothallic, with two mating types Mat A and Mat B, and natural isolates are in most cases haploid [10,11]. The mating frequency of two natural Mat-compatible strains is very low, but the resulting diploid state is stable under laboratory growth conditions [12]. Such hybrids exhibit a very low fertility, a problem that was alleviated through inbreeding programs to allow the establishment of the first genetic maps [10]. High sporulation rates can be obtained on peculiar media (yeast extract/malt extract or V-8 juice media, media with 1.5% sodium citrate as sole carbon source) and the shapes of asci and ascospores exhibit some strain-dependant variations [9]. Wild-type isolates of *Y. lipolytica* can present a large variety of colony aspects, ranging from smooth and glossy to strongly wrinkled and mat. This diversity reflects the fact that *Y. lipolytica* is a dimorphic yeast that can grow either as round multipolar budding cells, pseudohyphae (budding cells remaining attached) or mycelia with septate hyphae, depending on growth conditions [10,11,13]. This possibility of growth under different forms (dimorphic switch) is of practical importance regarding biotechnological applications of *Y. lipolytica*, since monitoring all environmental parameters will be crucial for the control of cellular morphology, from which the optimization of the bioprocess could depend on [14].

#### 2.2.3. Carbon Sources

*Y. lipolytica* is able to use as carbon source a large array of substrates of either hydrophilic or hydrophobic nature [10,11]. Water-soluble carbon sources include only a few sugars (glucose, fructose, mannose) but also glycerol and, to a lesser extent, organic acids and alcohols. The long-prevailing belief that *Y. lipolytica* could use only some hexoses, but no pentose, as sole carbon source has however been recently undermined in experiments on xylose assimilation by some strains, as will be reported in Section 2.3.3 and Section 3.2. Similarly, the rather recent discovery of a wild-type strain able to metabolize lactose has undermined the previous belief that this sugar could not be a substrate for *Y. lipolytica* (cf. Section 2.3.3 and Section 3.3.3). Water-insoluble carbon sources comprise fatty acids, triglycerides and alkanes. Remarkably, the engineering of *Y. lipolytica* metabolism for the use of alternative substrates has been initiated very early in its history of genetic manipulation: heterologous expression of *Saccharomyces cerevisiae SUC2* gene was used more than three decades ago to confer the ability to grow on sucrose to some of the most used laboratory strains [15]. Wild-type *Y. lipolytica* isolates present a high potential for the valorization of liquid or solid wastes from various agricultural and industrial origins, notably crude or raw glycerol issued from biodiesel production processes (cf. Section 2.3.3 and Section 3.3.2). In addition, genetic engineering of this yeast for use of other (agro)industrial wastes as alternative substrates for white biotechnology applications has become an important and rapidly developing research field, as will be exemplified further in this review (cf. Section 2.3.3). This overall versatility that this yeast allows in the choice of possible substrates represents a valuable asset for the development of bioprocesses involving *Y. lipolytica*, especially those based on the valorisation of by-products or waste.

#### 2.2.4. Secretion Pathway

The two more prominent characteristics of *Y. lipolytica* are its very efficient secretion pathway and its outstanding lipid storage capacity. Consequently, this yeast has become a research model in the domains of protein secretion and lipid metabolism [16,17]. The study of vesicular protein secretion in *Y. lipolytica* has demonstrated that the translocation of the nascent protein into the endoplasmic reticulum (ER) was mainly co-translational, as in the secretion pathway of mammals [16]. This peculiarity, contrasting to the situation in *S. cerevisiae* and most yeasts for which secretion is mainly post-translational, allows *Y. lipolytica* to be very efficient in the folding and secretion of large and/or complex heterologous proteins and has contributed to its success as a heterologous production host [18,19,20]. In addition, *Y. lipolytica* is one of the few yeasts, with *Pichia pastoris*, which lacks an α-1,3-mannosyltransferase, a factor that limits the amount of excessive mannosylation of secreted heterologous glycoproteins and constitutes a valuable asset for the production of therapeutic proteins [21,22].

#### 2.2.5. Lipid Storage

As an oleaginous yeast, *Y. lipolytica* can naturally accumulate lipids up to 30 to 50% of the cell dry weight (CDW), depending not only on each wild-type isolate genetic background but also on the carbon source used and the growth conditions. This lipid accumulation can reach up to 90% of CDW through genetic engineering, in obese *Y. lipolytica* cells [17] (cf. Section 3.1.4). As regards growth conditions, the accumulation of lipids in this yeast is known for a long time to be favoured by nitrogen starvation [17]. Lipid storage in *Y. lipolytica* results from an effective de novo synthesis pathway for triacylglycerols (TAG) when sugars or similarly catabolized compounds such as polysaccharides or glycerol are used as carbon sources. It is however more remarkable when hydrophobic substrates are used, benefiting then from both an efficient uptake of lipids from the medium and an efficient *ex novo* synthesis pathway (biomodification) [23]. When grown on non-fatty substrates (such as crude glycerol) most wild-type *Y. lipolytica* strains are not able to accumulate high levels of lipids, even under nitrogen-limited conditions, since those produced during the early growth steps are submitted to degradation to the benefit of other compounds such as organic acids and polyols [24,25]. Thus, during growth of wild-type *Y. lipolytica* on glycerol in bioreactor, in repeated batch cultures, three succesive phases were identified: a biomass production phase, a lipogenic phase and a citric acid production phase [26]. There are only a few exceptions to this rule, such as notably the SKY7 isolate (cf. Section 3.3.3). Using double- or multiple-limitation media could however alleviate this problem, as was demonstrated for ACA-DC 50109 strain (cf. Section 3.3.2). In this regard, wild-type *Y. lipolytica* strains appear somewhat atypical among oleaginous yeasts, for which lipid content is usually less substrate-dependent [27]. The physiological response of *Y. lipolytica* cells to the presence of hydrophobic substrates (such as alkanes, fatty acids or oils) consists in the production of biosurfactants (notably liposan), in a hydrophobization of the cell membrane and in the formation of protrusions on the cell surface [16]. These protrusions correspond to the hydrophobic binding structures of an interfacial transport system, composed of several dozens of multimeric protein complexes, which facilitate the uptake of hydrophobic compounds from the environment [17,28,29]. The very efficient secretion of the extracellular lipase *LIP2* also contributes to the effective uptake of lipids by this yeast, through a reduction in molecular weight of the hydrophobic substrates. The lipase family has known an expansion in *Y. lipolytica*, as in most oleaginous yeasts, with a total of 16 lipase genes. The storage lipids of *Y. lipolytica* consist mostly of TAG and sterol esters, more than free fatty acids (FFA) and accumulate in a specialized subcellular compartment, the lipid body (LB). These lipids can comprise as high as 80% of unsaturated fatty acids, which present some valuable health benefits. Notably, *Y. lipolytica* is the oleaginous yeast with the highest known percentage of linoleic acid (LA), namely more than 50% [17]. The lipid metabolism of this yeast is of particular relevance for some major white biotechnology applications, such as the production of single-cell oil (SCO) and of biofuel and has been the subject of numerous reviews [17,23,28,30]. Interestingly, the lipid profile of *Y. lipolytica* SCO can be modulated through the use of different mixtures of low-cost fatty substrates in order to provide tailor-made lipids, as was demonstrated notably by the obtention of cocoa-butter substitute from stearin, with chemically hydrolyzed rapeseed oil as co-substrate, using the wild-type strain ACA-DC 50109 (cf. Section 3.3.2) [31,32].

#### 2.2.6. Genomic Organization

The first *Y. lipolytica* strain to be completely sequenced and fully assembled and annotated, E150, which will be described in detail in Section 3.1.2, constitutes the reference strain for genome structure studies. Its genome of 20.5 Mb comprises six chromosomes which sizes range from 2.6 to 4.9 Mb [33,34]. This genome size is almost twice those of most other yeasts, including *S. cerevisiae* (12 Mb) [34]. Several other genomic characteristics make *Y. lipolytica* clearly stand out from the crowd of other hemiascomycetous yeasts. Notably, the G/C content, of 49% in average and near 53% in the genes, and the proportion of intron-containing genes, of 15%, are strikingly higher than for other yeasts (respectively, 38%, 40% and 5% in *S. cerevisiae*) [34,35]. In contrast, the number of genes, although on the strong side of the range for hemiascomycetous yeasts, is not as high as may be inferred from the large genome size. Namely, *Y. lipolytica* totalizes 6703 genes, more than the 5807 ones from *S. cerevisiae* but less than the 6906 ones from *Debaryomyces hansenii*, which both have genomes of around 12 Mb [34].

Nine other *Y. lipolytica* strains of different genetic background have now been sequenced (cf. Section 3 and Section 4.5) and the already assembled genomes show some chromosomal rearrangements compared to the reference strain, despite a nearly constant genome size [36,37]. This is consistent with the previous observation, in karyotypic analyses, of an important polymorphism in the length of various chromosomes between different *Y. lipolytica* strains. Such a high level of chromosomal rearrangements between strains could explain the poor fertility that was observed for the hybrids [38].

Among yeasts, *Y. lipolytica* presents atypical ribosomal DNA units, with several rRNA gene clusters scattered on different chromosomes (six clusters on four chromosomes in E150). In addition, the 5S RNA gene is not included in those rDNA unit but present as separated copies scattered throughout the genome [38]. These characteristics, such as the co-translational secretion pathway mentioned above, are closer to those of mammals than to those of other yeasts, confirming the eccentric phylogenetic position of *Y. lipolytica* based on the comparison of 18S and 26S rDNA sequences [38]. Some expression vectors for *Y. lipolytica* genetic engineering make use of rDNA sequences as targeting elements for integration into the genome (cf. Section 4).

The first *Y. lipolytica* retrotransposon identified, Ylt1, was detected in the E150 genome; this element can only be found in a few wild-type isolates and in their derivatives, such as the genetically modified (GM) E150 strain (cf. Section 3.1.2). Ylt1 belongs to the Ty3/gypsy group and is bordered by unusually large (more than 700 bp) long terminal repeats (LTRs) termed zeta sequences, which can also be found as solo elements in the genome [39]. The numbers of Ylt1 and of solo zeta sequences present in a genome vary for each Ylt1-bearing strain but is of at least 35 copies for the retrotransposon and more than 30 copies for the solo LTRs [39]. A number of other retrotransposons have since been identified in other *Y. lipolytica* strains [36,40,41], but the presence of Ylt1 in a genome is relevant for some metabolic engineering strategies, since zeta sequences have been used as targeting elements in some expression vectors or cassettes, as will be explained hereafter (cf. Section 4.1.2) [42,43]. In contrast to *S. cerevisiae*, *Y. lipolytica* does not bear any retrotransposon of the Ty1/Copia group, which are usually abundantly found in eukaryotic genomes. Interestingly, the presence in some strains of several retrotransposons and LTR-like sequences near to RNA polymerase III-transcribed genes, which number is almost twice that in most other yeasts, seems to indicate that these retro-elements may have played an active role in the evolution of *Y. lipolytica* [36].

### 2.3. An Impressive Curriculum Vitae: Short Review of Past, Present and Future Y. lipolytica Uses

#### 2.3.1. Industrial Applications of Wild-Type or Traditionally Improved Strains

The high potential of *Y. lipolytica* for industrial applications has been exploited since more than 70 years, at first in the fields of biomass and valuable metabolites production, using proprietary wild-type isolates or traditionally improved strains (mutants, strains issued from hybridizations and crossings) [1,44,45]. In the 1950s, and until the oil crisis of the 1970s, the British Petroleum Company (BP, London, UK) applied the alkane-degrading capacities of this yeast to the production of single-cell protein (SCP) from crude oil. The product was commercialized under the name Toprina G, for livestock feeding, and prompted numerous studies on *Y. lipolytica* safety that led to the first GRAS (generally recognized as safe) notification for a process using this yeast. In the 1970s, Pfizer Inc. (New York City, NY, USA) applied *Y. lipolytica* to industrial citric acid production. This technology was repurchased in the 1990s by Archer Daniels Midland Company (ADM, Chicago, IL, USA), who is still (to the best of our knowledge) producing some citric acid from *Y. lipolytica*, using mostly corn or rape seed oil as substrates [1,44,45].

Recent studies of citric acid production by wild-type *Y. lipolytica* isolates takes more into account sustainability factors by employing new wild-type strains able to use glycerol as substrate, in order to utilize biodiesel-derived crude glycerol wastes [46] (cf. Section 2.3.3 and Section 3.3.2). The obtention of citrate mutants was shown to increase the citric/isocitric acid ratio that is usually low with most wild-type strains grown on raw glycerol [47]. More surprisingly, using acid whey as a sustainable waste-derived substrate for citric acid production by a wild-type lactose-positive *Y. lipolytica* strain has also been proposed [48] (cf. Section 3.3.3). The development of industrial production platforms for other organic acids, such as iso-citric acid, from wild-type or traditionally improved *Y. lipolytica* strains, is also an important research area, as reviewed recently [49].

Other nowadays applications of proprietary wild-type or traditionally improved *Y. lipolytica* strains include, in the field of food industry, erythritol production by Baolingbao Biology Co. (Yucheng, Shandong, China) and use of *Y. lipolytica* biomass as fodder yeast for farm and pet animals by Skotan SA (Chorzów, Poland), who also develop prebiotic/probiotic applications [1,50].

The outstanding capacity of *Y. lipolytica* for degrading hydrocarbons, and especially alkanes, explains that wild-types isolates were frequently found in oil-polluted environments and justifies the use of this yeast in bioremediation projects [44]. Even though most of such research are only at the laboratory level, two field studies were reported in Poland: the wild-type *Y. lipolytica* A-101 strain (cf. Section 3.3.1) has been successfully applied to in situ depollution of petroleum-contaminated soils at a fuel base [51] and of creosote-contaminated soils at a timber processing plant [52]. In the same field of bioremediation, a *Y. lipolytica* based starter for depolluting wastewaters is commercialized by Artechno (Isnes, Belgium). This product corresponds to freeze-dried *Y. lipolytica* cells and secreted lipase from selected highly lipolytic mutants of ATCC 48436 strain (cf. Section 3.2), obtained using chemical mutagenesis at INRA [53]. In addition to bioremediation, other potential applications of wild-type *Y. lipolytica* strains include their use as biocontrol agents and eco-friendly biofertilizers in agriculture, or for immune-stimulation in aquaculture (shrimps, fishes), as highlighted in a very recent review focusing on *Y. lipolytica* ecological significance [7].

#### 2.3.2. Commercial Applications of Genetically Modified Strains

In the 1980s, the newly developed technics of molecular biology rejuvenated the interest in *Y. lipolytica*, this time as an expression host for producing heterologous proteins [18]. Metabolic engineering of this yeast ensued rapidly, following the development of transformation methods, shuttle vectors and non-leaky non-reverting auxotrophic strains [15]. As *Y. lipolytica* started, in the 2000s, to be recognized as a valuable host for recombinant protein production [19,20], the YLEX kit for expression/secretion of heterologous proteins in this yeast was commercialized in 2006 by Yeastern Biotech Co. (Taipei, Taiwan—http://www.yeastern.com/ (accessed on 3 June 2021)). This kit includes vectors for expression/secretion and the Po1g strain, a GM derivative of W29 wild-type isolate, obtained at INRA (cf. Section 3.1.3). During the same period of time, *Y. lipolytica* other W29 derivatives have been established as commercial protein production platforms by Protéus (Sequens Group, Ecully, France) and Oxyrane UK (Manchester, UK), both also making use of INRA technologies [54].

With the continuous progress of genetic engineering technics, increasingly complex modifications of *Y. lipolytica* metabolism, such as the introduction of complete heterologous metabolic pathways, could be performed. Proofs of concept of the use of this yeast as cell factory for the production of valuable compounds or as arming yeast for bioconversion processes are abundantly reported in the scientific literature since a few decades [54,55,56]. However, most of the proposed applications for these GM *Y. lipolytica* strains remain, until now, only at an exploratory stage and are not developed further to the industrial stage. This matter of fact could be attributed at least in part to social acceptance issues concerning GM microorganisms, especially in the domain of food applications. Until now, only a few commercial or industrial applications of GM *Y. lipolytica* strains can be reported [1,45]. GM *Y. lipolytica* cell factories are presently used for industrial production of two kinds of food/feed additives: carotenoids and polyunsaturated fatty acids (PUFAs)-rich SCOs. The technology of carotenoids production by *Y. lipolytica* was developed by Microbia (Lexington, MA, USA) and then acquired by DSM (Heerlen, The Netherlands) [45]. The technology of PUFAs-rich SCOs production by a heavily engineered *Y. lipolytica* strain derived from the ATCC 20362 wild-type isolate (cf. Section 3.2) was developed by DuPont (Wilmington, DE, USA) and was more particularly applied to industrial production of ω-3 eicosapentaenoic acid (EPA)-rich products [57,58]. The use of GM *Y. lipolytica* for producing EPA-rich SCO was patented by DuPont (US2009/0093543A1) and the corresponding engineering work was described in detail a few years later [57]. Remarkably, the development of this first commercially viable technology platform using GM *Y. lipolytica* was achieved using only the classical genetic engineering tools available at the time [58]. Two commercial products were derived from this technology platform: EPA-rich SCO for food and EPA-rich *Y. lipolytica* biomass for feed applications. The EPA-rich SCO has been marketed, as dietary supplement for human consumption, under the name New Harvest^TM^, but only briefly (from 2010 to 2013) before the product was discontinued. New Harvest^TM^ was advertised as the first vegetarian alternative to fish-based ω-3-rich oils, with no mention of its GM nature. No confirmation of social acceptance issues concerning the use of a GM microorganism was given by the company, concerning the product withdrawal. Since 2010, the EPA-rich *Y. lipolytica* biomass is marketed, in joint venture with AquaChile (Puerto Montt, Chile), as an ω-3 feed supplement for “harmoniously raised” salmon Verlasso^TM^. DuPont research teams have also recently engineered the same ATCC 20362 strain for producing 2′-fucosyllactose (2′FL), the major human milk oligosaccharide (HMO), with the aim of developing a commercially viable cell factory platform for 2′FL and perhaps for other HMOs for infant formulas [59].

Another domain of successful applications for GM *Y. lipolytica* strains is the therapeutic use of recombinant enzymes: several enzyme replacement therapies (ERTs) based on this yeast are now marketed or on the edge to marketing stage [54,55]. The first of these ERTs was developed by Mayoly Spindler (Chatou, France), in partnership with INRA and AzurRx Biopharma, Inc. (Brooklyn, NY, USA/Langlade, France), from a GM *Y. lipolytica* strain overexpressing *LIP2* [60]: this recombinant extracellular lipase is currently applied for the treatment of exocrine pancreatic insufficiency, and under Phase 2 clinical trial for two other fat malabsorption diseases (cystic fibrosis and chronic pancreatitis). More recently, Oxyrane (Ghent, Belgium), starting from Po1d strain (a GM derivative of W29 wild-type isolate, obtained at INRA—cf. Section 3.1.3), has established a proprietary *Y. lipolytica* engineering platform able to produce recombinant glycoproteins, with the possibility of added mannose-6-phosphate (M6P) glycan residues [61], for treatment of different lysosomal storage diseases. The presence of M6P on therapeutic glycoproteins improves their internalization into the patient’s cells and addresses them to lysosomes, their targeted subcellular site of action. A recombinant human acid α-glucosidase produced in *Y. lipolytica*, OXY2810, is currently marketed for use as ERT in Pompe disease, in which glycogen accumulates in the patient’s tissues. A recombinant glucocerebrosidase (GCase) is in preclinical testing for treatment of Parkinson’s disease and a GCase adorned with M6P glycans is in development for treatment of neuronopathic Gaucher disease, due to glucocerebroside accumulation in neuronal cells [54,55]. Oxyrane also envision to apply their *Y. lipolytica* platform to developing new ERTs targeting other metabolic diseases.

#### 2.3.3. Towards a Bio-Based Economy: Rewiring Strain Metabolism for Alternative Substrates

**Figure 1:**
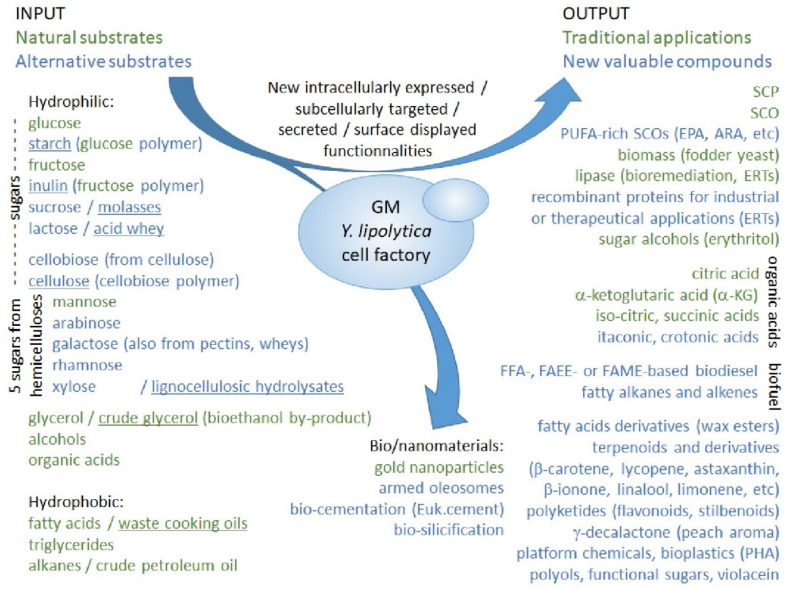
Schematic representation of genetically engineered *Y. lipolytica* cell factories as a generic “black box”, with exploitable substrates as input and potential applications and products as output.

As mentioned above, numerous proofs of concept of the use of GM *Y. lipolytica* for various potential white biotechnology applications can be found in the scientific literature. Their number is increasing rapidly: a search about “Yarrowia AND engineering” on PubMed website generates 17 results for the year 2010, an annual average of 30 results for the four following years, then an annual average of 64 results for the three following ones and finally an annual average of 105 results for the three last years, until 2020. For 2021, the search already generates 47 results already for the first trimester, which could be extrapolated to nearly 200 results if the rhythm of publication is maintained. These innovations have been abundantly described in many recent reviews [45,50,54,55,56,62,63,64,65] and will only be resumed here and schematically depicted in Figure 1 that represents a state of the art for substrates availability and biotechnological applications for GM *Y. lipolytica* strains.

Natural substrates and traditional applications of wild-type *Y. lipolytica* strains are indicated in green. Alternative substrates and new applications, requiring metabolic remodelling of *Y. lipolytica*, are indicated in blue (pentoses are indicated in blue, according to the fact that most *Y. lipolytica* strains cannot use them as sole carbon source: cf. more details in Section 2.3.3 and Section 3.2; lactose is also indicated in blue since only one wild-type isolate, B9, has recently been reported to be able to metabolize this dairy sugar: cf. Section 3.3.3). Substrates issued from agricultural, forest and industrial waste or by-products, corresponding to an environmentally friendly concept of circular bioeconomy, are underlined. Data compiled from several recent reviews [45,50,54,55,56,62,63,64,65]. Abbreviations used, per order of occurrence in the figure: GM, genetically modified; SCP, single cell protein; SCO, single cell oil; PUFA, poly-unsaturated fatty acids; EPA, eicosapentaenoic acid; ARA, arachidonic acid; ERTs, enzyme replacement therapies; α-KG, α-ketoglutarate; FFA, free fatty acids; FAEE, fatty acid ethyl esters; FAME, fatty acid methyl esters; PHA, polyhydroxyalkanoates.

The use of alternative renewable substrates as cheap carbon sources is aimed at valorizing waste or by-products from various human activities or industries, in a sustainable development and circular bio-economy approach. The possibility to use raw materials as feedstock is also of major importance for reducing the production cost, in order to allow the industrialization of processes that are still at the laboratory-scale (cf. Section 4.5.3 for some examples of such bioprocesses). Thus, the high potential of wild-type *Y. lipolytica* isolates for the valorization of liquid or solid wastes represents a valuable asset in the development of cleaner and more sustainable bioprocesses, in a circular bio-economy approach.

Crude or raw glycerol is the main by-product of biodiesel production and is also produced by other industries (oleochemistry, fat saponification, stearin, bioethanol or alcoholic beverages production sites). Despite its frequent contamination by salts, metallic ions and methanol, this by-product constitutes a favorite substrate for wild-type *Y. lipolytica* strains (notably for ACA-DC 50109 strain, as detailed in Section 3.3.2). This renewable feedstock can thus be converted into valuable compounds: mainly citric acid and polyols, but also lipases, SCO and even yeast biomass that could be reused in food and feed [66]. Other possible feedstocks for *Y. lipolytica*-based bioprocesses include olive mill wastewater, palm oil mill wastewater or waste cooking oil, together with fruit/vegetable, fish/sea food and meat wastes. Rather recently, glucose-containing wastes (starch and cellulose hydrolysates issued from farming and agro-food industries) have been proposed as interesting feedstock for citric acid production from wild-type *Y. lipolytica* strains (notably for K57 strain—cf. Section 3.3.3) [67]. Dairy wastes (acid whey) can also be partially processed by wild-type strains (as a nitrogen, vitamins or minerals source), but constitute a preferred feedstock only for the B9 isolate, the sole *Y. lipolytica* strain able to grow on lactose (cf. Section 3.3.3) [48]. The valorization of various wastes by wild-type *Y. lipolytica* strains has been the subject of two very recent extensive reviews (both presently online ahead of print) that demonstrate the economical and ecological importance of this yeast as a waste bioprocessing mmicroorganism [66,68].

Metabolic remodelling of *Y. lipolytica* for the use of alternative substrates is expected not only to improve the processing of waste or by-products already metabolized by wild-type strains, but also to extend this possibility of valorization to a much wider range of possible feedstocks issued from various human activities or industries. In addition to the use of crude glycerol and of waste cooking oils (recycled from fast-food and other catering establishments), which both are natural substrates for *Y. lipolytica*, genetic engineering can in addition allow the use of lignocellulosic hydrolysates from agriculture, forestry and paper industry, of starch- or inulin-rich agricultural wastes, of molasses from sugar industry and push further that of acid whey from cheese and yogurt industries [62,63,69]. Interestingly, the Wroclaw University of Environmental and Life Sciences (UPWr, Poland) collaborated with INRA for the design of GM *Y. lipolytica* strains combining an obese phenotype with a wide substrate range [70]. Notably, these teams engineered GM derivatives of either W29 or A-101 wild-type isolates to produce at first obese strains with increased lipid biosynthesis and storage, and then to add several biomass-derived sugars (galactose, fructose, sucrose and inulin) to the range of substrates that these strains can effectively use (cf. more details on the resulting strains, YLZ150 in Section 3.1.4 and Y4779 in Section 3.3.3). The W29-derived YLZ150 strain, which can perform very efficient lipid biosynthesis from a wide range of biomass-derived sugars, is a valuable chassis for SCO or biofuel production from non-lipid renewable resources. In contrast, the A-101-derived Y4779 strain would be more adapted to developing sustainable processes for citric acid or polyhydroxy alcohol production [70].

However, a major challenge for white biotechnology is to select or engineer microorganisms of interest that can efficiently metabolize xylose, the main component from lignocellulosic material. Indeed, lignocellulosic hydrolysates, which constitute the preferred substrate in a circular bioeconomy, are a raw mixture of nutrients, notably pentoses and hexoses (mainly xylose and glucose), originating from the degradation of cellulose and hemicellulose from plant biomass. These hydrolysates can also contain significant amounts of some compounds, such as organic acids and furfurals, which are toxic for most organisms but are rather well tolerated by *Y. lipolytica* [71]. This yeast has been regarded for a long time as unable to metabolize xylose, since the usual wild-type isolates were not able to use this pentose as sole carbon source. This drawback has prompted different research teams to engineer *Y. lipolytica* strains for xylose use, with the aim to develop sustainable SCO production platforms, as reviewed previously [71]. However, xylose consumption has rather recently been found to be strain-dependent (cf. Section 3.2) and omics studies have revealed a dormant pentose pathway in *Y. lipolytica* [72]. A recent collaborative work performed mainly at INRA has demonstrated that the overexpression of three genes from this dormant pentose pathway, in a Po1d genetic background, conferred to the recombinant strain the ability to grow on xylose as well as the parent strain on glucose, and was even more efficient than the overexpression of corresponding heterologous genes [71]. The same overexpression of native genes, performed in an obese derivative of Po1d strain, generated a strain able to accumulate lipids up to 67% of CDW, using xylose as sole carbon source. These two strains were also demonstrated to be able to grow efficiently in lignocellulosic hydrolysates and could potentially be applied to sustainable production of xylitol and citric acid, or of SCO/biofuel, respectively [71]. In order to achieve the same goals of developing sustainable industrial processes, the University of Texas at Austin (UT Austin-Austin, TX, USA) has recently constructed GM strains able to use xylose as sole carbon source, by addition of a heterologous oxidoreductase pathway, followed by a starvation adaptation step, into a Po1f strain and one of its obese derivatives, the E26 strain (cf. Section 3.1.4). The resulting XUS (xylose utilizing strains) were submitted to genome resequencing which revealed notably a duplication of the newly introduced *XYL1* and *XYL2* genes from *Scheffersomyces* *stipitis* to be a major factor for improved xylose consumption [73]. The PO1f XUS and E26 XUS strains were subsequently used in a strain mating strategy that allowed to combine their xylose consumption property with the capacity to synthetize various valuable compounds from several GM strains, as will be detailed in Section 4.4.2 [74].

#### 2.3.4. Potential Applications of Genetically Modified Strains: Bioproducts and Biofuels

In addition to the traditional biotechnological applications of *Y. lipolytica*, most of which have already been upgraded to industrial level (cf. Section 2.3.1), genetic engineering now allows to envision the production of a large range of valuable compounds. In addition, the production of natural metabolites or products of interest can also benefit of strain improvement obtained through metabolic engineering, either for increased yield or for use of alternative substrates, as has been exemplified for α-ketoglutaric acid (KGA) [75], for γ-decalactone (peach aroma) [76], for SCP [77] and for SCO [78], which composition can also be enriched in health-promoting ω-3 or ω-6 rich PUFAs, as seen above [57,58] (cf. Section 2.3.2) and reviewed previously [27]. The production of recombinant proteins for industrial or therapeutical applications represents an important research field that has been extensively reviewed previously [18,19,20,21,22]. The particular need to obtain more human-compatible recombinant glycoproteins for possible use as therapeutic agents has prompted the development of glyco-engineered (aka humanized) *Y. lipolytica* strains, which will be evoked in Section 3.1.6. In the domain of pharma/food industry, new metabolites of interest that can be produced using GM *Y. lipolytica* cell factories comprise notably a large range of terpenoids (including various carotenoids) and their derivatives [79,80,81], polyketides (including flavonoids) [80], riboflavin [74] and human milk oligosaccharides [59] (cf. Section 2.3.2). More recently, *Y. lipolytica* has also been applied to the production of a bacterial compound, violacein, a bis-indol purple pigment derived from the tryptophan pathway, which has numerous therapeutic, notably anticancer, properties [82]. GM *Y. lipolytica* are also considered as emerging cell factories for the production of several organic acids non-naturally produced by this yeast, of sugar alcohols (polyols) and of a number of functional sugars (including the naturally produced erythritol) which have a wide range of applications in different economic sectors [83,84,85]. In the chemical industry domain, GM *Y. lipolytica* are an interesting source of several value-added bioproducts: wax esters used as advanced lubricants [86]; polyhydroxyalkanoates and polylactic acid homopolymer used as bioplastics [87,88]; platform chemicals, such as itaconic and crotonic acids (organic acids) [82,89], ricinoleic acid (an unusual fatty acid) [90], triacetic acid lactone [74] or multi-purpose long chain dicarboxylic acids used notably in the synthesis of polyamides and polyesters copolymers [91].

Last but not least, GM *Y. lipolytica* cellular factories can constitute a particularly interesting source of biofuels, due to their high oleaginicity, to the possible use of inexpensive renewable carbon sources and to their robustness and good performance under stress, as was abundantly reviewed previously [92,93,94,95]. Increasing the oleaginicity of *Y. lipolytica* in order to obtain GM obese strains that can be used as chassis for producing various valuable lipid-related products and biofuels is an important research domain that will be reviewed in Section 3.1.4. Most research on *Y. lipolytica*-produced biofuel focus on rewiring its metabolism for an increased accumulation of fatty acids and derivatives, since FFAs, fatty acid ethyl esters (FAEEs) and fatty acid methyl esters (FAMEs) can be used directly as biodiesel. Alternatively, another strategy is aimed at the production of hydrocarbon-based biofuels. Notably, *Y. lipolytica* was engineered to obtain the biosynthesis of pentane through heterologous expression of a soybean lipoxygenase [96] and of larger odd-chain alka(e)nes through heterologous expression of the fatty acid photodecarboxylase from *Chlorella variabilis* [97,98]. Such studies demonstrate that GM *Y. lipolytica* cellular factories can also be used to produce renewable alkanes and alkenes.

Both of these engineering strategies, for fatty acid-based and hydrocarbon-based biofuel production, can benefit of recent methods of subcellular targeting of new functionalities to different cell organelles. A research team from the Massachusetts Institute of Technology (MIT-Cambridge, MA, USA) engineered *Y. lipolytica* into a yeast biorefinery platform for sustainable production of fuel-like compounds and oleochemicals, in which subcellular targeting of heterologous enzymes to the cytoplasm, the peroxisome and the ER allowed to generate FAMEs and fatty alkanes with tailored chain length [99]. Similarly, a team from the Huazhong University of Science and Technology (Wuhan, China) has targeted lipase dependent pathways to different lipid-related subcellular compartments (LB, peroxisome and ER), which allowed a substantial increase of FAMEs, fatty alkanes or fatty alkenes titers, compared to their cytosol-targeted engineered counterparts [100]. The same research team has also used an innovative method for the in vivo self-assembly of multienzyme complexes (MECs) in order to improve fatty acid-derived hydrocarbon production in *Y. lipolytica* cells. Their strategy was based on the simultaneous surface display of scaffoldin, a synthetic multiple cohesins backbone and extracellular secretion of several dockerin-fused heterologous enzymes implied in the new metabolic pathway [101]. The highly specific interaction of cohesin and dockerin domains allowed the spontaneous assembly of MECs carrying up to three enzymatic functions, in a combinational way. Namely, different proportional and positional effects could be genetically encoded in a customized scaffoldin backbone by varying the copy number and orientation of the cohesin domains used. This methodology allowed an optimization of the co-immobilized enzymes efficiency, through a substrate channelling effect that generated a 17-fold enhancement in their initial reaction rate. The resulting MECs exhibited much higher conversion yields (71–84%) in alka(e)ne production than an equivalent cocktail of free enzymes (8–32%) [101].

#### 2.3.5. Potential Applications of Genetically Modified Strains: Nanoparticles and Biomaterials

A more unexpected domain of applications of GM *Y. lipolytica* cells is the production of biomaterials or nanostructures [54]. Previously, some wild-type *Y. lipolytica* strains had been applied to the biosynthesis of metallic nanoparticles for biomedical applications, notably NCIM 3589 strain for gold nanoparticles (AuNPs) production [102]. As the formation of AuNPs was directed by the melanin/pyomelanin produced by *Y. lipolytica* cells, a more recent strategy consists in engineering *Y. lipolytica* strains for enhanced production of these pigments that could then be purified and applied to AuNP synthesis. Notably, the synthesis of AuNPs mediated by pyomelanin purified from a GM W29 strain was recently reported, and a multifactorial statistical analysis of the process parameters allowed to fine-tune the size of these nanoparticles for different medical applications, such as imaging or drug delivery [103].

Prior to its use for subcellular compartment engineering, the technic of oleosome targeting had been developed by a research team of Hawaii University at Manoa (Honolulu, HI, USA) for the purpose of designing tunable functional nanoparticles [104]. Surface display of different heterologous proteins on *Y. lipolytica* LB was obtained through fusion with a heterologous oleosin from plant and the resulting subcellular structure was fractioned through sonication into nano-oleosomes of tunable size. The obtained armed oleosomes correspond to stable nanoparticles (200–300 nm of diameter) used as scaffold for protein display, which can be equipped with various functionalities and used for numerous biomedical applications such as biosensors, cell targeting or drug delivery [104]. This work has also brought the first proof-of-concept that co-expressing in *Y. lipolytica* different heterologous proteins fused with either a dockerin or a cohesin domain would lead to their in vivo self-assembly that, combined with their surface display on oleosomes, can generate functional nanofactories applicable to various biotechnological purposes [104].

Surface display of silicatein on the surface of GM *Y. lipolytica* cells has been used by a research team of Ocean University of China (Qindao) to aggregate armed yeasts into flocs, a sheet-like biosilica-yeast hybrid material, for bioremediation applications [105]. At last, an ambitious and complex engineering work, directed by Huazhong University of Science and Technology (Wuhan, China), led to the design of Euk.cement, an autocementation kit project based on live GM *Y. lipolytica* cells. Euk.cement is expected to stabilize underwater sands by biologically induced autocementation, for civil engineering or environmental restoration purposes. Surface-display and secretion of recombinant peptides and proteins by GM *Y. lipolytica* cells lead firstly to their immobilization onto silica sand particles and secondly to carbonate sedimentation, namely autocementation [106]. The Euk.cement kit project has received a gold medal at iGEM (International Genetically Engineered Machine) Competition in 2015 (MIT, Cambridge, MA, USA—http://2015.igem.org/Team:HUST-China (accessed on 3 June 2021)) but is not, to the best of our knowledge, commercialized yet.

### 2.4. Long-Lost Relatives: Other Yeasts of the Yarrowia Clade

#### 2.4.1. Brief Outline of Phylogeny, Habitat and Characteristics

*Y. lipolytica* has a fairly chaotic phylogenetic history: in its early days as a yeast species, only the anamorph (i.e., asexual state) was known and classified as *Candida lipolytica*. The teleomorph (i.e., sexual state) was discovered only in 1970 and classified as *Endomycopsis lipolytica*, before being renamed *Saccharomycopsis lipolytica* and, finally, *Yarrowia lipolytica*, as summarized previously [1]. For more than 50 years, *Y. lipolytica* has been considered as the only species of the *Yarrowia* genus and this is only for two decades, when sequencing has become a routine technic for taxonomic studies, that other species have been identified. The comparison of sequences from D1/D2 domains of the large subunit (LSU) rRNA gene and from internal transcribed spacer regions (ITS1 and ITS2) has highlighted some heterogeneity among former *Yarrowia* isolates and has also shown that some yeasts previously belonging to the catch-all asexual *Candida* genus needed to be reattributed to the *Yarrowia* clade [107,108,109,110,111,112,113]. The sequencing of the mitochondrial DNA of four Candida species (*C. alimentaria*, *C. deformans*, *C. galli* and *C. phangngensis*) has also confirmed their belonging to the *Yarrowia* clade [114]. In addition to *Y. lipolytica*, 12 other *Yarrowia* species are now listed on the NCBI Taxonomy Browser webpage (https://www.ncbi.nlm.nih.gov/Taxonomy/Browser/wwwtax.cgi (accessed on 3 June 2021)).

Most of these new species have been isolated in Europe, USA or Asia, from a large range of natural habitats, rather similar to those of *Y. lipolytica*: dairy and meat food products for *Y. alimentaria*, *Y. oslonensis*, *Y. divulgata*, *Y. galli*, *Y. bubula* and *Y. porcina* [1,107,108,112,113]; mangrove or marine waters for *Y. keelungensis*, *Y. phangngensis* [1,109,110] and also for other strains of *Y. divulgata* [112]; guts of insects or vertebrates for *Y. parophoni*, *Y. yakushimensis* and *Y. hollandica* [1,108,111]. More unexpectedly, *Y. deformans* was isolated from humans, notably from a fingernail [111], which could raise concerns about its innocuousness (as is also the case for some *Y. galli* isolates [115]. At last, a new species has been proposed more recently for addition to the *Yarrowia* clade, *Y. brassicae*, isolated from a fermented vegetal food, Chinese sauerkraut [116]. These different species of the *Yarrowia* clade have in common to be Crabtree-negative oleaginous yeasts, to grow through multilateral budding, to be able to form both pseudohyphae and true hyphae, to possess an expanded lipase family (7 to 16 genes) and to be physiologically rather similar (e.g., use of a limited number of sugars and polyols as carbon sources) [117]. A few differences were however observed in substrate use: for example, *Y. brassicae* present the unique characteristic of growing on inulin, an interesting asset for potential applications [116]. The maximum growth temperature of these yeasts is rather variable, from 27 °C for *Y. alimentaria* to 37 °C for *Y. phangngensis* [117]. Another oleaginous yeast, *Candida hispaniensis*, has been often associated with an extended version of the *Yarrowia* clade, although this affiliation remained dubious [117]. Published phylogenic trees of yeasts from the *Yarrowia* clade, established from D1/D2 LSU rRNA gene sequences, identify *Y. yakushimensis* as the species closest to *Y. lipolytica* and *Y. alimentaria* as the furthest one in the *Yarrowia* genus, with *C. hispaniensis* being still further, at a basal position [108,116,118]. More recent data, obtained from further study and full genomic sequencing of *C. hispaniensis*, clarified the phylogenic position of this oleaginous yeast outside from the *Yarrowia* clade, as a close parent of the genus [119]. The term “extended *Yarrowia* clade” will however continue to be used in this review for earlier studies still including *C. hispaniensis*. This phylogenic revision is consistent with the fact that many characteristics of *C. hispaniensis* differentiate this yeast from all the members of the *Yarrowia* clade (no (pseudo)hyphae, compact genome of 10.6 Mb, G/C content of 41%, lipase family reduced to 3 genes) [119]. Nevertheless, *C. hispaniensis* presents some particularly attractive characteristics for industrial biolipid production (30% more oleaginous than *Y. lipolytica* W29 strain, faster replication, no filamentation, no competing citric acid production) and the proof of concept of its amenability to genetic engineering has been very recently demonstrated [119]. A more recent study, focused on variations in the telomeric repeats (encoded in telomerase RNAs—TERs) and in the repeat-binding proteins from all species (except *Y. brassicae*) from the extended *Yarrowia* clade confirmed the homogeneity of the *Yarrowia* genus and the external position of *C. hispaniensis* [120]. This work determined a 10 bp consensus sequence for the TER-derived telomeric repeat unit of all *Yarrowia* species, with minor variations between species, and identified in all of them a functional homologue of *Y. lipolytica* Tay1 protein able to bind all repeat variants. This study provided significant insights into the co-evolution of TERs, telomeric repeats and telomere-binding proteins in the *Yarrowia* clade and, more generally, in yeasts [120].

#### 2.4.2. Potential Applications of Other Yeasts of the *Yarrowia* Clade

There have only been a few reports of exploring the biodiversity of the different yeasts from the *Yarrowia* clade for biotechnological applications [117,118,121], and a single attempt of engineering one of them with the tools developed for *Y. lipolytica* has been reported in the scientific literature [122]. An INRA study compared growth, lipid synthesis and storage of *Y. lipolytica* and 8 other yeasts from the extended *Yarrowia* clade, during growth on 31 non hydrophobic (sugars, carbohydrates) and 13 hydrophobic (triglycerides, FFA, alkanes) carbon sources. Despite a common oleaginicity, the specific patterns of substrate use and lipid storage varied: for example, the lipid content of cells grown on oleic acid ranged from 30% of CDW for *Y. oslonensis* to 67% for *C. hispaniensis* [121]. In collaboration with the UPWr (Wroclaw, Poland), *Y. lipolytica* and 11 other yeasts from the extended *Yarrowia* clade were tested for erythritol, mannitol or citric acid production from pure glycerol, glucose or fructose. If only *Y. lipolytica* was able to secrete citric acid, several alternative *Yarrowia* strains were able to produce efficiently polyols, including erythritol and mannitol. The best sweetener producer was *Y. oslonensis*, while *Y. hollandica* and *Y. divulgata* could also be promising species, provided optimizing media composition and cultivation parameters [117]. Research teams from the National Center for Agricultural Utilization Research (USDA-ARS, Washington, DC, USA) investigated biomass and lipid production capacity of 57 strains of the extended *Yarrowia* clade (45 *Y. lipolytica* isolates and 1–2 isolates of 12 other species) grown on a low-cost renewable feedstock (non-detoxified dilute acid-pretreated switchgrass hydrolysate), in order to expand the diversity of oleaginous yeasts amenable for bioeconomy [118]. With the exception of three of them that were unable to grow in these conditions (*Y. alimentaria*, *Y. yakushimensis* and *C. hispaniensis*), most alternative *Yarrowia* strains were able to accumulate lipids as well or better than the reference W29 *Y. lipolytica* strain, at least for the longer growth times. Two of them, the *Y. phangngensis* and *Y. hollandica* type strains, were top lipid producers with maximum lipid titers, respectively, fourfold and two-fold higher than W29 [118]. A subset of interesting strains has been further characterized for inhibitor tolerance, production kinetics and fatty acid composition, for intended future biotechnological applications.

Interestingly, the same USDA-ARS teams have recently demonstrated that several genetic tools designed for use in *Y. lipolytica* could also be applied to *Y. phangngensis* engineering, with little or no need for modifications [122]. These authors used some promoters, expression vectors and antibiotic resistance genes from *Y. lipolytica*, applied a similar transformation protocol and finally employed the Cre-lox system for marker recycling designed for this yeast (cf. Section 4.2.5), in order to engineer the PT1–17 type strain of *Y. phangngensis* for improved lipid production from cellulosic feedstock (acid-pretreated switchgrass hydrolysate). The GM *Y. phangngensis* strain exhibited a 58% decrease in lag time and a 32% increase in lipid titer compared to the parent wild-type strain, due to enhanced detoxification of inhibitor compounds and to added carbon flux into the triacylglycerol synthesis pathway [122]. This demonstration of the easy amenability of an alternative *Yarrowia* strain to genetic engineering is expected to generate a new interest in the highly oleaginous *Y. phangngensis* species and can be hoped to be a good omen for the other yeasts of the *Yarrowia* clade.

## 3. Fantastic Yeasts and Where to Find Them: *Yarrowia* Strains and Yeast Collections

The main purpose of this Section is to provide a useful tool for both advanced users and beginners in *Yarrowia* biotechnology, by reviewing the main *Y. lipolytica* strains of interest with their characteristics and usual domains of application, and by identifying the main yeast collections that makes them (and other species of the *Yarrowia* clade) publicly available worldwide. The Table 1 presents a selection of *Y. lipolytica* wild-type or GM strains of industrial/biotechnological interest, a few of which will be described in more detail below. Only the strains that have been deposited in at least one yeast collection have been listed in this Table, which makes them easily available for research purposes (at the exception of a few of them, as indicated). Other strains cited further below in this Section should be requested directly from the corresponding laboratories.

The different culture collections worldwide that preserve and provide these strains are listed in Table 2, with their website address and their standard of qualification (ISO standard). The total number of strains from *Y. lipolytica* species present in each collection is also indicated, together with that of less identified *Yarrowia* sp. and of other species of the *Yarrowia* clade. This Table is intended as a useful tool to ease the identification, for a given research team, of the closer or more appropriate source for *Yarrowia* strains in general and for precise remarkable strains in particular. For the sake of readability and considering the low number of existing samples of each of the alternative *Yarrowia* species, which makes their search easier, their reference numbers in each collection have not been indicated. The type strain of *Y. phangngensis* that has been the basis for the first engineering assays of alternative *Yarrowia* species described in Section 2.4.2 [122], PT1–17, was deposited at the USA ARS (NRRL Y-63743) and at the Dutch CBS-KNAW (CBS 10407) yeast collections. These two institutions are the more complete sources of alternative species of the extended *Yarrowia* clade in the world; besides *Y. lipolytica*, all 14 other species can be found at CBS-KNAW and 12 of them at ARS (at the exception of *Y. parophoni* and of the more recently described *Y. brassicae*).

### 3.1. Oldies but Goodies: Elder Y. lipolytica Strains and Their Derivatives

The different *Y. lipolytica* strains described in this Section that are publicly available can be found, for the most widely used of them, in different yeast collections (cf. Table 1 and Table 2), but the CIRM-Levures INRAE yeast collection (France) is the only one that can provide all of them.

#### 3.1.1. From Paris Sewer to Worldwide Renown: The Success Story of W29

**Figure 2:**
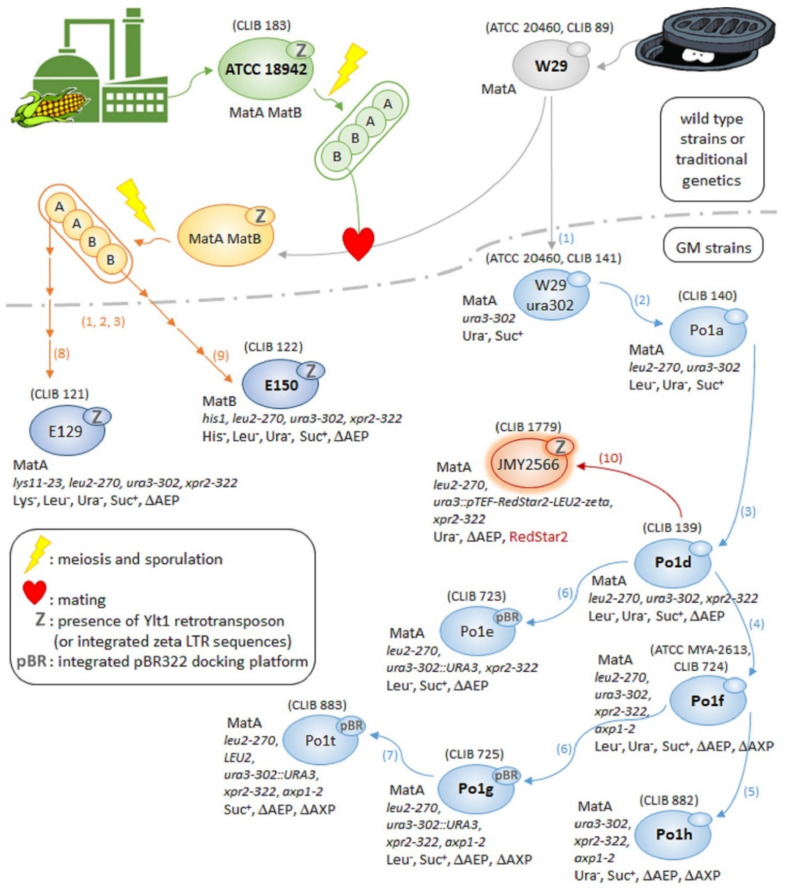
Genealogy of strains of biotechnological interest derived from ATCC 18942 and/or W29. Schematic representation of the origins of ATCC 18942 and W29 wild-type strains and their publicly available derivatives: genotypes and phenotypes are indicated near each strain; yeast collection reference numbers are shown only for ATCC and CLIB-Levures (others can be found in Table 1). Genetic events are represented by the lightning and heart symbols; the presence of sequences that can be used for targeting integrations (zeta sequences, plasmidic backbone) is indicated in the nascent yeast bud. The genetic engineering steps are indicated by bracketed numbers, as follows: (1) genic conversion of *URA3* into *ura3-302* (corresponding to *ura3::*p*XPR2:SUC2*, namely to a 728 bp *Xho*I-*Eco*RV deletion in *URA3* coding region with disruption by the *SUC2* gene from *S. cerevisiae* under the control of the *XPR2* promoter, conferring the ability to grow on sucrose or molasses) [15]; (2) genic conversion of *LEU2* into *leu2-270* (681 bp *Stu*I deletion in *LEU2* coding region); (3) genic conversion of *XPR2* into *xpr2-322* (149 bp *Apa*I deletion in *XPR2* coding region, inactivating alkaline extracellular protease); (4) genic conversion of *AXP1* into *axp1-2* (655 bp *Nco*I deletion in *AXP1* coding region, inactivating acid extracellular protease); (5) genic conversion of *leu2-270* into *LEU2* (restoration of wild-type allele); (6) integration of *Nco*I-linearized pINA300’ plasmid (*URA3* gene in pBR322 [173]); (7) integration of *Not*I-linearized *LEU2*-carrying pINA1269 plasmid [159] at pBR docking platform; (8) genic conversion of *LYS11* into *lys11-23*; (9) genic conversion of *HIS1* into *his1*; (10) genic conversion of *ura3-302* into *ura3::pTEF-RedStar2-LEU2-zeta* (replacement of the *SUC2* expression cassette disrupting *URA3* with two cassettes, for expressing the RedStar2 fluorescent protein from *TEF* promoter and for *LEU2*, and a zeta LTR sequences docking platform) [162]. The names of the more prominent strains are in bold type. Information gathered from [10,43] or ATCC and CLIB-Levures websites.

The W29 wild-type strain has been isolated in the 1970s from sewage water, in Paris (France) [154], and attracted attention for its robustness of growth and high capacity of secretion of various enzymes (proteases, lipase, RNase) [10,155]. It became rapidly the favourite choice for developing heterologous protein production tools at INRA and was implied in the French *Y. lipolytica* strain inbreeding program [10,11]. W29 is the most ubiquitous strain in yeast collections worldwide and has become a sort of reference for comparing the performances of new *Y. lipolytica* isolates or of alternative *Yarrowia* species for growth or production of various compounds [118]. The genetic background of W29 has benefited to its various GM derivatives, which constitute since decades the most frequently used strains in the domain of heterologous protein production [18,19] (cf. Section 3.1.2 to Section 3.1.6). This strain has also been applied to organic acid production [11]. Its genome is the third to have been fully sequenced and assembled [36,156], after those from E150 and Po1f (see below), although these strains were its progeny/derivatives. The mitochondrial sequence of W29 had however been determined earlier [172]. The sequencing strategy used and the *de novo* assembly of its genome have been the first to enable obtaining a single contig for each of the six chromosomes A to F and for the mitochondrial chromosome M [36]. In contrast to the reference sequenced strain E150, W29 is a strain devoid of Ylt1 retrotransposon as well as of solo Ylt1 LTRs (zeta sequences, used as targeting elements in some vectors—cf. Section 4.1.2). Several other genomic structure variations and differences in the presence of retro-elements were observed in the parent W29, compared to the progeny E150, notably the inversion of a 71 kb fragment from the chromosome B and the presence of a novel Ty3/Gypsy retrotransposon with multiple associated LTR-like sequences [36]. The sequencing and assembly of the wild-type W29 strain also provided data on *Y. lipolytica* rDNA sequences (which were excluded in previous genomic analyses) together with complete sequences for *URA3*, *LEU2*, *HIS1* and *XPR2* genes, which carried deletions in previously sequenced auxotrophic strains [36,156]. The main GM strains derived from W29 will be described below, with their genealogy represented in Figure 2.

#### 3.1.2. W29 and ATCC 18942 Progeny: E129 and E150 Strains

The early French/USA *Y. lipolytica* strain inbreeding program aimed at establishing genetic maps and developing new strains through mating of isolates of industrial interest, notably W29 and ATCC 18942, this latter being issued from a corn-processing plant in the USA. The ATCC 18942 strain, sometimes designated as YB-423 from its NRRL reference number, is the *Y. lipolytica* diploid type strain [138], exhibits as such a particularly robust growth and has been notably applied to yeast biomass production [139]. As already said in Section 2.2.2, the mating frequency of two natural Mat-compatible strains is very low and more recent sequencing data, highlighting the surprisingly large difference in the presence of retrotransposons and other retro-elements between strains (see above), could constitute an explanation to this long-dating observation.

Nevertheless, as schematized in Figure 2, the sporulation of the diploid ATCC 18942 strain was performed and a Mat-compatible ascospore was selected and used for mating with W29. The resulting diploid was then subjected to sporulation in order to generate ascospores of both mating types, from which multiple steps of genetic engineering allowed to design the “sister” GM strains E129 and E150, of compatible mating types [10]. The parent ATCC 18942 strain bearing naturally Ylt1 retrotransposon, the derived E129 and E150 strains also contain numerous copies of this retrotransposon, as well as solo zeta sequences, that are available to be used as multiple dispersed targeting elements for further engineering (cf. Section 4). E129 and E150 are both tri-auxotrophic strains that carry the *ura3-302* allele, corresponding to a disruption of *URA3* gene by a heterologous cassette expressing *ScSUC2* that confers them the ability to metabolize sucrose [15]. They are also deleted for the major secreted protease, AEP (alkaline extracellular protease, expressed at neutral/alkaline pHs), encoded by the *XPR2* gene, a characteristic useful for applications in heterologous protein production [10]. The E129 strain has been one of the early strains to be applied to heterologous protein production, but it has been since abandoned in favour of direct derivatives of W29, which secretion system appeared to be more efficient in coping with high increases of secretory pathway cargo load in multicopy-expressing strains [155].

The E150 strain was selected in the French Génolevures II Project [34] to become the first *Y. lipolytica* genome to be fully sequenced, essentially on the basis that, being issued from a crossing between French and USA isolates, it would carry all the different retrotransposons that had already been identified in these *Y. lipolytica* strains at this time. Therefore, E150 now represent the reference strain for the assembly and annotation of *Y. lipolytica* genomes. However, when performing sequence homology searches, it is to be remembered that E150 is a GM strain, carrying deletions in several genes (*URA3*, *LEU2*, *HIS1* and *XPR2*) and containing an added heterologous gene (*SUC2* from *S. cerevisiae*).

Being issued from a meiosis event, the mating type-compatible GM strains E129 and E150 have related, if not identical, genetic backgrounds. Their triple auxotrophy could permit engineering them using *URA3* and *LEU2* as selection markers and to cross them while selecting for diploid formation on minimal medium, thanks to the remaining auxotrophies for lysine or for histidine, respectively, in E129 and in E150. Mating between these “sister” strains occurs at a satisfactory, if not optimal, frequency (C. Madzak, unpublished results) and the resulting diploid could cumulate the genetic modifications and present an improved robustness (cf. Section 4.4.2 for implementation of similar mating strategies).

#### 3.1.3. W29 Derivatives: The Po1 Series of Strains

The favourable genetic background of W29 for protein secretion has prompted the design at INRA of a series of GM derivatives for applications in the domain of heterologous protein production. As represented in Figure 2, W29 has been at first equipped with an *ura3-302* allele (*URA3* disrupted by *ScSUC2* cassette) that provided both an auxotrophy and the ability to use sucrose as sole carbon source [15]. Then, a series of “Po1” GM strains where derived, by addition of leucine auxotrophy, by deletion of the major (AEP) or of both extracellular proteases (AEP and AXP) and finally by complementation of one or of both auxotrophies [18,19]. The Po1d strain, bearing two auxotrophies due to non-reverting mutations and able to metabolize sucrose [158], remained for more than a decade the most frequently used recipient strain worldwide for heterologous protein production. Po1d is notably the host strain used as chassis in the design of the ERT-producing platform from Oxyrane (Ghent, Belgium) (cf. Section 2.3.2) [61].

The privilege of being the most frequently used host has now been transmitted to the Po1f strain [159], which is additionally deleted for the second *Y. lipolytica* extracellular protease (AXP, expressed at acidic pHs, in contrast to AEP), thus eliminating all secreted proteasic activity that could be a threat for heterologous proteins. Po1f is the second *Y. lipolytica* strain to have had its genome fully sequenced and assembled [160,161], which reflects the recognition of its predominance as a metabolic engineering host [18,19,20]. In contrast to the other Po1 strains, which can be obtained only from CIRM-Levures yeast collection, Po1f can also be found at ATCC and VKPM (cf. Table 1 and Table 2).

In contrast to Po1d, f and h strains, which can retain the GRAS status of *Y. lipolytica* since their engineering brought only yeast-derived sequences, the strain Po1g has been equipped with an integrated bacterial-derived sequence, namely a pBR322 docking platform, in order to facilitate the further integration of pBR322-based expression vectors. This easy-to-use integration system takes benefit of the large region of homology between these vectors and the docking platform to obtain very high transformation efficiencies (in the range of 10^4^ to 10^5^ transformants per μg of DNA) and a high percentage of targeted integration (in the range of 80–90%) despite the high level of non-homologous end joining (NHEJ) in *Y. lipolytica* cells [43,159]. The Po1g strain has been included in the Yeastern YLEX commercial kit (cf. Section 2.3.2), together with two pBR322-based vectors, one for intracellular expression and one for secretion of heterologous proteins. This strain retains only a leucine auxotrophy, allowing its transformants to be prototrophs. The YLEX kit was aimed at easy and rapid testing of heterologous production of a given protein in *Y. lipolytica* and has also been demonstrated to be particularly adapted to enzyme engineering, notably through directed mutagenesis [174,175]. Namely, targeting to the pBR322 docking platform allows to obtain the integration of a unique copy of the heterologous cassette at a precisely known neutral locus in the genome, so that the effect of the different mutations tested can be directly compared by measuring the recombinant enzymatic activity of the various transformant strains. Interestingly, the Po1g strain has been chosen to constitute the basis of Cell Atlas, a series of seven isogenic strains in which different organelles were rendered fluorescent by tagging with GFP (green fluorescent protein from *Aequorea victoria*), designed by a consortium of research teams from Richland (Richland, WA, USA) for the purpose of cell biology applications [176].

Other strains of the Po1 series include Po1h, which retains only an uracile auxotrophy, and the prototrophic Po1t strain, which was designed to serve as a negative control when testing heterologous production of a protein in one of the other Po1 strain. At last, a tri-auxotrophic derivative of Po1f strain has also been recently designed at UT Austin, by addition of a tryptophan auxotrophy [177]. This new strain, named PO1j (PO1f *trp1*::*loxP*) has not, to the best of our knowledge, been deposited in any public yeast collection. Similarly, a tri-auxotrophic derivative of Po1d strain, Po1dh, deficient for 5-aminolevulinate synthase, has been constructed to allow the use of an artificial chromosome strategy (cf. Section 4.1.4).

#### 3.1.4. Other Derivatives of W29: Obese Strains

Lipid accumulation in *Y. lipolytica* cells result from a complex pattern of interactions between different metabolic pathways, in different cell compartments: lipid synthesis involves mainly the cytosol, lipid storage the ER and the LB and lipid mobilization the LB and the peroxisomes. The different strategies used for increasing lipid accumulation in several *Y. lipolytica* strains using metabolic engineering have been extensively reviewed very recently [178] and will only be briefly evoked below. The compilation of all previous works indicated not only that W29 and its derivatives have been the most used for this purpose, but also that the W29 genetic background allowed to design the best obese GM strains [178]. Several research teams through the world have used different strategies of genetic engineering to push further the oleaginous potential of W29 derivatives in order to obtain GM strains with up to 75% or even 90% of lipids in their CDW [179,180,181]. Such obese strains constitute precious tools as powerful chassis that could be applied to the production of various valuable lipid-related products and biofuels.

In addition to enhancing lipid storage capacity, genetic engineering of *Y. lipolytica* strains can also result in breaking free from the constraint of using only fatty substrates (*ex novo* synthesis pathway, biomodification) that limits the industrial use of most wild-type strains for SCO production. In particular, GM strains can be engineered for using crude glycerol as substrate for lipid accumulation through *de novo* synthesis pathways. The main strategies used for constructing obese strains include: (i) inactivating degradation pathways, notably the *β*-oxidation pathway, by knocking-out *POX* genes [179,180,182,183]; (ii) increasing the availability of precursors for lipid biosynthesis, notably of glycerol-3-phosphate (G3P), through different mutations in the G3P shuttle pathway (knocking-out the *GUT2* gene for anabolic dehydrogenase, or overexpressing *GPD1* and/or *GPD2* genes for catabolic dehydrogenases) [179]; (iii) overexpressing genes implicated in lipogenic metabolic pathways, notably *DGA1* and *DGA2* for diacylglycerol acyltransferases [181,184]; (iv) knocking-out some enzymes which inactivation could mimic nitrogen starvation conditions, notably *PHD1* for 2-methyl-citrate dehydratase [180,185]; (v) boosting the cytosolic redox metabolism through NADH to NADPH conversion [186].

The first strategy chosen at INRA consisted in deleting several genes from the glycerol-3-phosphate (G3P) shuttle pathway and the β-oxidation pathway, in order to increase the availability of G3P and of FFA that both constitute limiting factors for TAG synthesis. This first attempt at improving *Y. lipolytica* oleaginicity allowed to engineer Po1d into an obese strain (Po1d Δ*gut2*Δ*pox1-6*) that can accumulate lipids up to 75% of CDW [179]. More recently, as briefly evoked in Section 2.3.3, the UPWr (Poland) collaborated with INRA, at first to develop some obese strains from either a W29 or an A-101 genetic background, and then to engineer them further for use of a wide substrate range [70]. These teams engineered at first Po1f to generate the Y4086 obese strain (Po1f Δ*pox1–6*, Δ*tgl4*, *pTEF-DGA1*, *pTEF-GPD1*, *pTEF-YlHXK1*, *pTEF-ScSUC2*), with the ability to use sucrose (heterologous expression of *ScSUC2*) and an improved use of fructose (overexpression of hexokinase *HXK1*) [183] and, then, the ability to use other alternative substrates was added through overexpression of the endogenous Leloir pathway (*GAL* genes, for galactose use) [187] and through heterologous expression of the *Kluyveromyces marxianus INU1* inulinase gene (for degradation of inulin into fructose). The resulting YLZ150 obese strain was demonstrated to be able to efficiently use glucose, fructose, galactose, sucrose and inulin for lipid biosynthesis, with the highest lipid concentration (24 g/L) obtained from inulin [70]. The W29 genetic background appeared to be more appropriate for efficient lipid biosynthesis in these experiments than the A-101 one (see more details on A-101-derived obese strains with increase substrate range in Section 3.3.1), with a lipid concentration that was 57% higher [70]. The YLZ150 obese strain, able to use a large range of renewable biomass-derived sugars for SCO or biofuel production, paves the way to a sustainable bioeconomy.

Independently, an ambitious strategy of combinatorial strain engineering from UT Austin allowed an extensive rewiring of the lipid metabolic pathways of Po1f that generated an obese strain with the best lipid level ever reported, of almost 90% of CDW, with a lipid titer of 25 g/L [180]. The UT Austin research team approach combined multiplex inactivation of two genes (in β-oxidation and peroxisome biogenesis pathways) and overexpression of six lipid synthesis target genes, from three distinct metabolic pathways, with phenotypic induction to generate a series of obese strains. Their results allowed to advance fundamental understanding of lipogenesis in *Y. lipolytica*, by demonstrating notably that it can be uncoupled from nitrogen starvation and is dependent on leucine-mediated signalling [180]. The resulting obese strain with the highest performance (Po1f *pex10*, *mfe1*, leucine^+^, uracil^+^, *DGA1* overexpression) exhibits a de novo lipid accumulation more than 60-fold higher than its parent Po1f strain and has a high industrial potential for SCO and biofuel production [180]. This obese strain was further improved through an evolutionary engineering approach consisting in reiterated steps of random mutagenesis followed by floating-based selection, taking advance of the increased buoyancy brought to the cells by an enhanced lipogenesis. One of the improved obese strain obtained, E26, exhibits a saturating lipid content of 87% of CDW [188] and was used in the UT Austin strain mating project combining xylose assimilation to valuable compounds production (cf. Section 2.3.3 and Section 4.4.2) [74]. Full genome sequencing of the evolved strains revealed a link of the obese phenotype with *uga2* (succinate semialdehyde dehydrogenase) mutations, suggesting an unexpected role of γ-aminobutyric acid assimilation in lipogenesis [188].

At last, a MIT research team reported the highest carbon to lipid conversion yield (85% of theoretical maximal) and lipid yield titer (55 g/L) ever obtained from *Y. lipolytica* [181]. This MIT obese YL-ad9 strain has been obtained, from Po1g strain, by simultaneous overexpression of three genes (*ACC1*, *DGA1* and *SCD*), including a δ-9 stearoyl-CoA desaturase gene which product was identified as rate-limiting by reverse engineering from a mammalian cell obese phenotype [181]. Unexpectedly, this YL-ad9 obese strain demonstrated a growth rate threefold higher than its *LEU2*-complemented parent Po1g strain, which explains its productivity and reinforces its competitiveness for developing a robust and effective process for producing biodiesel or other lipid-derived compounds.

#### 3.1.5. Other Derivatives of W29: High-Throughput Expression Platforms for Protein Engineering

The design of efficient enzyme-based industrial processes generally require an optimization of the desired enzymatic properties through protein engineering and/or directed evolution. Some adapted host strains for expression, and high-throughput systems for screening, are required to respond to this technological demand. To fulfil such a purpose, the first *Y. lipolytica* high-throughput expression platform has been developed, nearly 15 years ago, as the result of a collaboration between INRA, INSA Toulouse and Toulouse University (Toulouse, France). The host strain used in this platform, JMY1212, has a W29 genetic background: it was derived from MTLY60 strain, a Po1d derivative deleted for extracellular lipases (Po1d Δ*lip2*, Δ*lip7*, Δ*lip8*) which was equipped with an integrated zeta docking platform [189]. MTLY60 and JMY1212, both intended for lipid metabolism engineering purposes, have not been deposited, to the best of our knowledge, in any yeast collection. As explained above for Po1g and its pBR322 docking platform, the zeta docking platform of JMY1212 allows the targeted integration of a unique copy of any zeta-based vector, which facilitates the screening of new or improved enzymatic functions [189]. In addition, the fact that zeta-based plasmids are auto-cloning vectors (integration of a cassette devoid of bacterial sequences—cf. Section 4.1.2) can result in the retention of the GRAS status of *Y. lipolytica*. The JMY1212 high-throughput expression platform was notably applied to engineering *Candida antarctica* lipase B (CalB), a widely used enzyme in industrial biocatalysis, for which it allowed the construction of large libraries of CalB mutants and the screening of new variants with a higher catalytic efficiency [190].

More recently, another high-throughput expression platform has been designed at INRA, based on the JMY2566 strain (also a Po1d derivative), as described in Figure 2 and in Table 1. JMY2566 retains only an uracile auxotrophy and, such as JMY1212, is equipped with an integrated zeta docking platform that eases both its transformation with any zeta-based vector and the ulterior screening of enzymatic properties [162]. In contrast to JMY1212, JMY2566 can secrete lipases but is deleted for the extracellular AEP protease. In addition, JMY2566 also bears a cassette for constitutive expression of a red fluorescent protein, RedStar2, which can be used as reporter for cell growth in non-translucent media, during the screening steps [162]. The JMY2566 recipient strain can notably be used as host for the Gateway overexpression vector JMP1529, using an optimized high-throughput transformation method in 96-well plates, so that the platform can be applied to high-throughput mutant library screening. Alternatively, this platform can also be applied to functional exploration of *Y. lipolytica* genetic regulation, as demonstrated by the recent analysis of 148 putative transcription factors by a systematic overexpression approach [191].

#### 3.1.6. Other Derivatives of W29: Glyco-Engineered Strains for Producing Therapeutic Proteins

The differences existing between the N-glycosylation pathways in yeasts and in mammalian cells can pose problem for the production of therapeutic proteins. Namely, glycoproteins produced in yeasts display high mannose-type N-glycans that could reduce the in vivo half-life of proteins and render them immunogenic in humans and other mammals. To palliate this problem, numerous research teams throughout the world have developed glyco-engineered (aka humanized) strains in the yeast species currently applied to heterologous production [22]. Concerning *Y. lipolytica*, a consortium of Belgian laboratories from VIB (Vlaams Instituut voor Biotechnologie) and Ghent University, together with Oxyrane (Ghent, Belgium), have constructed several glyco-engineered strains able to produce more human-compatible glycoproteins, from W29 and one of its derivatives constructed at INRA, MTLY60 (cf. Section 3.1.5). Notably, glyco-engineered strains able to produce glycoproteins homogeneously carrying Man5GlcNAc2 residues were obtained by the deletion of a two yeast-specific mannosyltransferases and heterologous expression of a fungal mannosidase [21]. Similarly, the mannosyltransferase deleted strain was further engineered by overexpression of a glucosyltransferase and heterologous overexpression of a fungal mannosidase and a fungal glucosidase, which generated a strain able to produce glycoproteins homogeneously carrying Man3GlcNAc2 residues, a core structure common to the different mammalian N-glycans that can additionally be modified in vitro to generate any kind of complex-type N-glycan [192]. In addition to the Oxyrane engineering platform able to add M6P residues to glycoproteins, for the development of ERTs for lysosomal storage diseases [61] described in Section 2.3.2, these glyco-engineered strains could contribute to upgrade *Y. lipolytica* into a valuable source of recombinant proteins carrying humanized N-glycans structures compatible with therapeutic applications.

#### 3.1.7. The Outsider H222

The H222 wild-type strain has been isolated in the 1980s from soil samples, in Leipzig (Germany), and attracted attention for its robust growth on fructose as sole carbon source, markedly better that those of other *Y. lipolytica* strains, and its high level of citric acid production [10,144]. H222 has been mainly applied to organic acid production and has been genetically engineered at the Technische Universität Dresden (TUD, Dresden, Germany), in collaboration with INRA, to design GM strains with improved citric acid production and able to metabolize sucrose (with the *ura3-302* allele bringing a *ScSUC2* cassette) [145,146]. TUD also collaborated with Helmholtz-Zentrum für Umweltforschung (UFZ, Leipzig, Germany) to engineer H222 for improved production of succinic acid or KGA [193,194]. The robust growth of H222 on fructose was demonstrated to be due to a better fructose assimilation, compared notably to W29 [183] and a study of *Y. lipolytica* sugar transporters explained further the superiority of the H222 strain in this domain [195]. Notably, one of the functional fructose transporters of H222 was found to exist only as a silent pseudogene in W29, and there was some amino acid polymorphism in eight hexose transporters between the two strains, which could account for the better fructose uptake of H222 [63]. Even though the subject of previous genomic studies [33,34], the H222 strain has been fully sequenced and assembled only recently [37]. This *de novo* genomic assembly of H222 revealed important differences with the reference strain E150, notably three major chromosomal rearrangements (two reciprocal translocations and a 300 kb inversion) that explain the observed variations in chromosome sizes [37]. This work illustrates the importance of preferring *de novo* genomic assembly over reference-assisted scaffolding for *Y. lipolytica* and highlights the important chromosomal rearrangements that can be found between strains.

### 3.2. Gold Diggers: How to Find Nuggets in Old Mines

The yeast collections listed in Table 2 constitute a valuable resource of biodiversity in the search for new *Y. lipolytica* genetic backgrounds and some of them have been noticed to be particularly adapted for peculiar applications, as shown in Table 1. For example, as said above in Section 2.3, two wild-type isolates from the US ATCC collection, ATCC 20362 and ATCC 48436, have been selected for commercial applications. The strain ATCC 20362, also referred to as 2002 strain in some publications, is an USA isolate that was cited in the US patent 3856667A from Bioteknika International Inc., in the 1970s, for applications in the degradation of petroleum crude oil. It was selected much more recently by a Dupont research team, for its robustness and oleaginicity, and applied to the design of their GM PUFA-producing platform (cf. Section 2.3.2) [57,58]. The strain ATCC 48436, an isolate from Japanese soil samples, has been noticed in the 1960s for its high levels of lipase activators and lipase activity [140], which prompted its more recent selection by INRA for the development of highly lipolytic mutants [53], currently applied to bioremediation by Artechno (cf. Section 2.3.1). Similarly, a tropical marine strain from the Indian NCIM Collection, NCIM 3589, has been studied for hydrocarbon degradation, biofilm formation and emulsifier production [147,148,149] before being used for the production of gold nanoparticles [103], which have applications notably in medicine (antibacterial and anticancer properties), in some diagnosis tests and in biomedical imaging.

At last, a consortium of USA laboratories from the National Center for Agricultural Utilization Research (USDA-ARS, Washington, DC, USA) performed a wide screening of the biomass and lipid production capacity of 45 *Y. lipolytica* wild-type isolates from the USA NRRL (ARS) collection under industrial type conditions, namely using a non-detoxified dilute acid-pretreated switchgrass hydrolysate as low-cost biomass feedstock [118]. This study identified five promising candidate strains, NRRL YB-392, YB-419, YB-420, YB-566 and YB-567, which genetic backgrounds are well adapted for industrial use, demonstrating better biomass hydrolysate consumption, inhibitor tolerance and lipid production than the W29 reference strain under those conditions. Notably, the three best strains (YB-392, YB-419 and YB-420, from which only the first two appear to be publicly available) produced from 43 to 64% more lipids than the W29 reference strain. These three strains can grow efficiently on 90% undetoxified switchgrass hydrolysate and convert cellulosic sugars into SCO with a fatty acid profile similar to rapeseed (canola), hence suitable for biodiesel production. Two other strains (YB-566 and YB-567, not publicly available) can assimilate xylose more efficiently than usual *Y. lipolytica* strains and convert cellulosic biomass into sugar alcohols such as xylitol or arabitol [118]. This work has also contributed to revise some inexact notions about pentose assimilation in *Y. lipolytica*: the fact that this yeast cannot use xylose as sole carbon source does not imply that its pentose assimilation pathway is missing or incomplete. In fact, as reviewed in their publication, xylose (and arabinose) consumption following glucose depletion was reported and bioinformatics tools demonstrated that *Y. lipolytica* has a functional xylose pathway, which enzymes are poorly expressed and for which xylitol dehydrogenase constitutes a limiting step [72]. Among the 28 *Y. lipolytica* strains (on the 45 tested) that were able to exhaust glucose in the hydrolysate, there was a surprisingly large variability (of a tenfold range) in the ensuing xylose consumption [118], which illustrates the interest of exploring strain biodiversity when searching for peculiar capacities. The five strains with high potential for industrial biocatalysis selected in this study have been recently sequenced [171], with the aim to allow further improvement of their robust metabolism by activating and/or rewiring pathways for improved complex sugar assimilation and lipid accumulation.

### 3.3. Finders Keepers: For a Good Isolate, Help Yourself

Some research teams have preferred to developed applications from wild-type *Y. lipolytica* strains they have themselves isolated, generally from oil-polluted soils or from marine environments and selected in general for a particular type of bioprocess. Among the numerous strains of this Section, only those deposited in at least one yeast collection are show in Table 1. The strains not publicly available from a collection should be requested directly from the corresponding laboratory.

#### 3.3.1. A-101 and Derivatives

In the 1990s, a research team from the Academy of Agriculture of Wroclaw (Poland) isolated the strain A-101 from oil-polluted soil samples from a car wash site, obtained some UV-generated mutants from it and compared these strains to a selection of ATCC *Y. lipolytica* strains. A-101 exhibited a robust growth on oil and a high citric acid production, which was enhanced in some mutant derivatives [123,124]. As described in Section 2.3.1, A-101 was also applied with success to in situ soil bioremediation [51,52]. This strain was deposited at the yeast collection of the UPWr (Wroclaw, Poland) but is not publicly available. More recently, A-101 has become a metabolic engineering host for the design of improved strains producing citrate or erythritol from various substrates, in the context of collaborations between UPWr and INRA [70,126,127]. Notably, some derivatives of this strain were engineered for highly efficient sucrose consumption throught an optimized expression/secretion of the heterologous *ScSUC2* invertase [126]. A draft genome sequence of A-101 has been made available, which interestingly revealed that this strain carries, among multiple retro-elements, a single copy of a solo zeta sequence (LTR of Ylt1) [125]. At last, as briefly evoked in Section 2.3.3, UPWr and INRA developed an obese strain from A-101 and engineer it for using a wide range of substrate [70]. In order to minimize the number of genetic modifications required in this strain, the β-oxidation pathway was blocked by deleting the multifunctional enzyme gene *MFE2*. In order to improve TAG biosynthesis, glycerol-3-phosphate dehydrogenase (*GPD1*) and diacylglycerol acyltransferase (*DGA1*) genes were overexpressed. In order to improve fructose use, a hexokinase (*HXK1*) and an efficient hexose transporter (*YHT3*) were also overexpressed. At last, in order to allow inulin use, a heterologous *K. marxianus* inulinase gene was added [70]. The resulting Y4779 strain was specifically tailored for a better use of fructose and did not compared very favourably with obese strains derived from a W29 background (cf. Section 3.1.4), but it appeared to be particularly adapted to the development of sustainable processes for citric acid or polyhydroxy alcohol production [70]. Interestingly, the use at Wroclaw University of Environmental and Life Sciences (Poland) of a traditionally improved A-101 derivative for citric acid production unexpectedly led to the identification of a spontaneous mutant with increased erythritol productivity and provided some insight into erythritol assimilation in *Y. lipolytica*. During cultivation of the A-101.1.31 strain (obtained through UV mutagenesis of A-101) in a nitrogen-limited chemostat for continuous citric acid production from glucose, a spontaneous mutation generated the K1 strain that was identified as a better erythritol producer, due to its inability to utilize this compound once produced. Submitted itself to UV mutagenesis, the K1 strain generated a MK1 strain with lower by-product formation but that recovered the ability to metabolize erythritol [128]. Submitted to whole genome sequencing, this series of mutant strains revealed, in K1, the formation of a stop codon in the ORF (open reading frame) of a gene of previously unknown function and, in MK1, the reversion of this knocking-out mutation for a change in the encoded amino acid. The target gene was named *EUF1*, for Erythritol Utilization Factor, and its involvement in erythritol catabolism was confirmed through RT-PCR analysis and deletion/overexpression studies [128].

#### 3.3.2. ACA-DC 50109, Its Derivatives and Other Greek *Y. lipolytica* Isolates

The strain ACA-DC 50109, also referred to as LGAM S(7)1 in some early publications, has been isolated at the Agricultural University of Athens (AUA, Greece) and applied to citric acid and SCO production, thanks to its high lipid content and productivity [129,130,132]. This strain has been deposited for safe only at ACA-DC (cf. Table 1 and Table 2) but can be requested from the Laboratory of Food Microbiology and Biotechnology (AUA). ACA-DC 50109 has been used both to decipher lipid accumulation mechanisms in *Y. lipolytica* and to develop sustainable bioprocesses based on the use of (agro)industrial wastes as feedstock, at AUA and Patras University (Greece). As described in Section 2.2.5, the lipid profile of ACA-DC 50109 has been modulated through the use of peculiar substrates (such as a mixture of stearin and chemically hydrolyzed rapeseed oil) in order to obtain SCO with a composition similar to that of cocoa-butter, which could serve as a cheap and substainable substitute of this high-valued compound [31,32]. Thanks to its robust growth on crude glycerol, ACA-DC 50109 has been applied to the valorization of this abundant industrial waste for simultaneous production of high lipid and citric acid yields [131]. Olive mill wastewaters have also been successfully used as feedstock for the production of citric acid [132].

Rather recently, an unexpected role of magnesium in *Y. lipolytica* lipogenesis has been revealed in a study at the University of Patras, who explored the lipid accumulation mechanisms in ACA-DC 50109 strain under single or double limitation conditions. Even though neither nitrogen nor magnesium single limitations were able to substantially increase lipid production from this wild-type strain, a double nitrogen and magnesium limitation allowed the spectacular accumulation of more than 47% *w*/*w* in lipids, the highest yield reported for a wild-type isolate of *Y. lipolytica* [130]. More recently, the same research team was able to improve lipid accumulation in ACA-DC 50109 strain through an adaptative laboratory evolution strategy that used alternatively environments promoting cell growth or lipid storage, in order to select oleaginous lineages of high energy-containing cells. After 77 generations under these lipid-prone directed evolution conditions, an evolved strain containing 44% *w*/*w* of lipids (a 30% higher level than its ACA-DC 50109 parent strain) was obtained [135].

Genetic engineering of ACA-DC 50109 has been initiated at the Ocean University of China (OUC, Qingdao), after an uracil mutant of this strain was isolated using counter-selection on 5′-FOA (5′-fluororotic acid, a toxic uracil precursor), and this strain is used as metabolic engineering host for the design of improved GM strains, notably for SCO production from alternative substrates such as inuline [133,134]. Interestingly, a global analysis of the acetylproteome (lysine acetylation of proteins, a major post-translational modification) has been performed in this strain, at the OUC, which revealed that lysine acetylation sites were present in more than 22% of *Y. lipolytica* proteins [196]. Notably, lysine acetylation was demonstrated in 65 enzymes from the lipid biosynthesis pathways, which highlights the crucial role that reversible acetylation could play in *Y. lipolytica* and possibly in other oleaginous microorganisms [196].

In addition to ACA-DC 50109, the Laboratory of Food Microbiology and Biotechnology (Agricultural University of Athens, Greece) has isolated a large number of *Y. lipolytica* strains from different local foodstuffs (sourdough, olives, kefir, meat or fish). All these strains (with codes ACA-DC, ACA-YC and LMBF Y) are available on request to the laboratory. The ACA-DC 5033 wild-type strain (aka ACA-YC 5033 in the laboratory’s yeast collection), one of those isolated from acid wheat sourdough [136], exhibits a robust growth on crude glycerol and the advantageous capacity of being able to produce simultaneous high yields of citric acid and of lipids [24]. Proposed applications for this strain include the production of SCO, citric acid and polyols (mannitol, erythritol and arabitol) from biodiesel-derived crude glycerol [24,137]. Among the other food-derived isolates from this laboratory, the LMBF Y-46 strain, isolated from a Mediterranean fish (*Sparus aurata*), has demonstrated a remarkable capacity for growth on crude glycerol and production of high amounts of mannitol in a screening study involving 11 yeast strains from four genera (including six *Y. lipolytica* strains: ATCC 20460, ACA-DC 50109 and four other isolates of the laboratory) [197]. The cultivation of this LMBF Y-46 isolate on glycerol, under nitrogen limitation conditions, in flask, produced different sugar-alcohols (mannitol, arabitol and erythritol) as major metabolites, when only negligible amounts of citrate were produced [46]. However, surprisingly, a metabolic shift towards citric acid production was observed when the same strain was cultivated in bioreactor, with only insignificant amounts of polyols simultaneously produced. When cultivated in fed-batch bioreactor, LMBF Y-46 was able to produce up to 102 g/L of citric acid, a level among the highest reported for a wild-type *Y. lipolytica* strain [46]. The metabolic transition observed was attributed to a higher oxygen saturation level in bioreactor compared to flask cultivation. Concomitantly, the lipids produced in bioreactor were shown to contain a higher proportion of unsaturated fatty acids compared to those produced during flask cultivation [46]. In addition to the demonstration of the interesting industrial potential of LMBF Y-46 isolate, this study highlighted the drastic importance of fine-tuned cultivation conditions and the subtleties of their interactions with the cellular metabolism. Such metabolic shift effects were also observed with other wild-type strains cultivated in similar conditions. While cultivated in flask under nitrogen limitation, with crude glycerol as substrate, two food-derived isolates ACA YC 5029 and 5030 produced high yields of mannitol and erythritol. However, in constrast, when cultivated in submerged batch experiments, the same ACA YC-5029 strain produced mainly citric acid [198].

#### 3.3.3. New Kids on the Block: *Y. lipolytica* Strains Isolated or Noticed More Recently

The Unesco Chinese Center of Marine Biotechnology, at OUC (Qingdao, China), is an institution devoted at exploiting marine microorganism resources for white biotechnology. In the 2000s, their research team isolated 78 wild-type *Y. lipolytica* strains from various marine-related environments (seawater, algae, marine or saltern sediments, guts from marine fishes), which were screened for interesting properties, notably for crude protein content. Seven of these marine strains were shown to contain more than 41% of proteins per CDW, with SWJ-1b having the highest crude protein level [4]. This strain, isolated from the guts of a marine fish, was applied to SCP and citric acid production [150] and became a metabolic engineering host for the design of improved GM strains [151], notably able to metabolize inulin [152]. SWJ-1b Can be obtained from the Marine Culture Collection of China (MCCC 2E00068). More recently, non-GM mutant strains were generated from SWJ-1b by a consortium of laboratories from Huaiyin Normal University and Institute of Technology (Huaian, China) [153]. These research teams used the recent atmospheric and room temperature plasma (ARTP) mutagenesis method, which constitutes a safer and more efficient alternative to traditional technics (e.g., UV, mutagens) allowing to generate mutations in the genome. One of these mutant strains, M53, exhibited the highest erythritol yield (0.65 g/g, from glycerol, in fed-batch fermentor) ever reported for *Y. lipolytica* or other microorganisms, which demonstrates its potential for erythritol production on a commercial scale [153].

The strain WSH-Z06 has been isolated rather recently, a decade ago, in the screening of 100 oil-polluted soil samples from 20 oil refineries from Wuxi (China) for KGA-producing yeasts, at Jiangnan University (Wuxi, China) [166]. This *Y. lipolytica* strain is auxotrophic for thiamine and a natural overproducer of KGA [166,167]. It has been deposited at the China Center for Type Culture Collection but is not publicly available. The WSH-Z06 strain has been applied to the production of KGA and of other keto acids [167]. This wild-type isolate has been sequenced and has been used as basis for a complex mutagenesis strategy (separate or combined use of ARTP with UV and mutagens) that generated a series of five hyper-producer mutant strains that were also fully sequenced [168]. This comparative genomics analysis provided insight into the physiology of KGA accumulation in *Y. lipolytica*, notably by highlighting a positive correlation with mitochondrial biogenesis and energy metabolism [168]. Interestingly, a proteomic analysis of the response of this strain to an acidic pH stimuli allowed a better understanding of the metabolic flux shift to KGA observed at lower pH, to be linked to its antioxidant role, and suggested process optimization strategies for short-chain carboxylate production [199]. At last, WSH-Z06 strain is also a metabolic engineering host for the design of GM strains improved for KGA production [169,170].

Until 2015, all known *Y. lipolytica* wild-type strains were reported to be lactose-negative; this long-prevailing belief has been negated by the discovery of the new B9 isolate in local soil samples, at the Ataturk University (Erzurum, Turkey) [200]. This new *Y. lipolytica* strain is able to metabolize lactose and can be applied to bioprocesses using acid whey as feedstock. Notably, a cold-adapted B9 strain has been used for citric acid production from partly deproteinized whey, in a process based on immobilized cells and non-sterile culture conditions [48]. This new lactose-positive B9 strain has been sequenced and its genome deposited into GenBank (access number KF486913).

The VKM Y-2373 and VKM Y-2412 isolates have been selected among several dozens of wild-type *Y. lipolytica* strains (and several different other yeast genera) for their remarkable performances in the production of, respectively, either citric or isocitric acids and KGA. VKM Y-2373 is a natural overproducer of isocitric acid when grown on rapeseed oil and of citric acid when grown on glucose, under nitrogen-limited conditions [163,164]. VKM Y-2412 is a natural overproducer of KGA when grown on rapeseed oil [165] or on crude glycerol (valorization of biodiesel waste) [201]. These strains, isolated at the Pushchino Scientific Center for Biological Research of the Russian Academy of Sciences (Moscow Region, Russia), are in safe deposit at VKM (cf. Table 2) but not publicly available from this collection. The VKM Y-2373 strain has been mutagenized using UV irradiation and chemical treatment to generate the 704-UV4-A/NG50 mutant that exhibits an improved production of citric/isocitric acids [163].

The SKY7wild-type *Y. lipolytica* strain has been isolated from a local forest soil sample at INRS-ETE (Institut National de la Recherche Scientifique / Eau, Terre et Environnement, Québec, Canada) [202]. The aim was to search for oleaginous microorganisms in a nitrogen-deficient soil environment, in order to select strains amenable to industrial lipid production. In contrast to most wild-type *Y. lipolytica* strains that are not able to accumulate high levels of lipids when grown on non-fatty substrates, even under nitrogen-limited conditions, since they degrade them to the benefit of organic acids and polyols, the SKY7 isolate is able to convert efficiently crude glycerol into triacylglycerides [203]. Due to its remarkable lipid production capacity (up to 42% *w*/*w*), the SKY7 isolate represents a very interesting candidate for lipid production using crude glycerol from biodiesel industry as sole carbon source. In addition, the lipid profile of the SCO produced by this strain is similar to that of vegetable oil, which makes it itself a possible feedstock for biodiesel production [203]. Thus, SKY7 appears as a valuable strain for improving waste-based sustainable circular economy bioprocesses.

The wild-type K57 strain, isolated at the Food Engineering Department of the University of Ankara (Ankara, Turkey) has been recently noticed as a promising candidate for processing of glucose-containing wastes (cf. Section 2.3.3). In a comparative study of 10 strains (wild-type isolates, including W29, as well as different H222 GM derivatives) for their industrial potential of citric acid production from glucose under nitrogen-limited conditions, K57 demonstrated the best performance. This strain was able to produce more than 72 g/L of citric acid from glucose, a level that outperform those of all known wild-type strains, thanks notably to its higher glucose uptake rate [67].

## 4. A Brave New World of Engineered Strains: Tools and Strategies for Building *Y. lipolytica* Cell Factories

**Figure 3:**
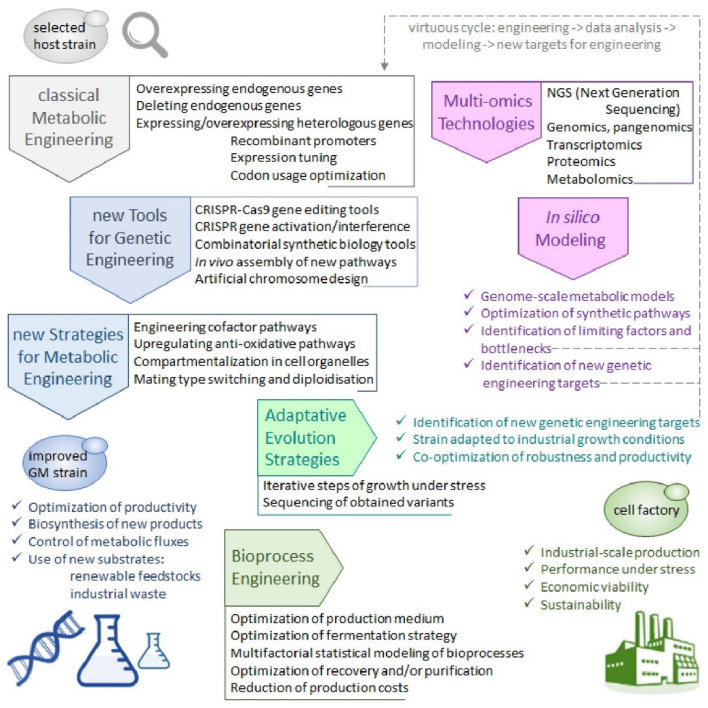
Schematic representation of the possible steps and available tools for engineering a *Y. lipolytica* strain into an effective cell factory. As explained in more detail in the text, both classical metabolic engineering methods and more recently developed tools, such as CRISPR-derived strategies, can be applied to remodelling metabolic pathways for production of a compound of interest. In addition, new strategies for metabolic engineering take also into account the availability of cofactors, the reduction of oxidative compounds and the compartmentalization of the modifications in different cell organelles, in a holistic view of the metabolic fluxes. The obtained GM (genetically modified) strain can also benefit of multi-omics technologies which, by allowing in silico modelling of genome-scale metabolic pathways, could contribute to identify limiting factors and bottlenecks, suggesting future genetic engineering targets in a virtuous circle. Adaptative evolution strategies could be employed to better adapt the GM strains to the stress of industrial growth conditions. At last, bioprocess engineering is required to develop viable and sustainable cell factories.

The process of transforming a selected *Y. lipolytica* host strain into a successful cell factory represents a long journey, through multiple technical steps requiring complementary expertises, that is tentatively schematized in Figure 3. As represented in this Figure, remodelling the metabolic pathways of *Y. lipolytica* for the production of a compound of interest can be obtained via deletion/repression/activation/overexpression of endogenous genes combined with (over)expression of a few heterologous genes as well as introduction of complete new metabolic pathways, all steps achieved through classical or more recently developed engineering/editing methods. The data gathered from different omics technologies could allow the development of genome-scale metabolic models that could influence the next engineering steps in a virtuous circle. Adaptative evolution strategies could also be applied for further improvement and, at last, bioprocess engineering will permit the valorisation of the laboratory achievements into an industrial-scale economically viable bioprocess.

Most of these processing steps have already been extensively reviewed previously [19,20,54,55,204,205,206,207,208,209] and this would be out of the scope of this publication to detail all of them, but a brief overview of the available genetic engineering tools will be presented, with a focus of those that could permit leverage of the natural biodiversity of *Y. lipolytica* strains and, possibly, of other yeasts from the *Yarrowia* clade.

### 4.1. To Be or Not to Be Integrated: Types of Vectors and Assembly Methods

Metabolic engineering of *Y. lipolytica* requires the assembly of single or multiple expression cassettes (or transcription units, TUs), each composed of a promoter, of an open reading frame (ORF) and of a terminator. In addition to the homologous or heterologous gene of interest, targeting components can be optionally included into the ORF, in order to direct the resulting recombinant protein to precise intracellular organelles or to the secretion pathway, either for release into the cultivation medium (vesicular secretion) or for display on the cellular surface (surface display). When classical genetic engineering strategies are used, TUs are carried by either integrative or replicative shuttle vectors, built and propagated in *Escherichia coli* strains, that are then introduced into *Y. lipolytica* cells rendered competent using chemical treatments [43,162,210] or electroporation [211,212].

The co-expression of several heterologous genes is generally needed when the introduction of entire metabolic pathways is required to confer new potentialities to *Y. lipolytica* strains. Vectors carrying multiple TUs can be used for that purpose, as reviewed previously [54,55] and resumed below. If some ambitious genetic engineering works have been performed using the classical (laborious and time-consuming) method of sequential integration [57], complex engineering projects can now benefit of new rapid in vitro or in vivo DNA assembly methods that were extensively reviewed previously [54] and will only be briefly evoked below in Section 4.1.3 and Section 4.1.4.

#### 4.1.1. Episomal Vectors

In order to be able to replicate in *Y. lipolytica*, an episomal vector needs to carry an ARS (autonomously replicating sequence)/CEN sequence, bearing co-localized replicative and centromeric functions. Such replicative vectors behave similar to mini-chromosomes, present in only one or a few copies per cell and easily lost during mitosis [213,214], which limit their interest for heterologous protein production. In contrast, they constitute the preferred tool for transient expression, as required for marker rescue using the Cre-lox system [215] or for gene editing using CRISPR tools (cf. Section 4.2.5 and Section 4.3.1). Replicative vectors have also been chosen for developing some of the recently designed genetic toolkits for *Y. lipolytica* pathway engineering, such as the YaliBricks system [216,217] (cf. Section 4.1.3). Interestingly, some engineering of a *Y. lipolytica* centromeric region by fusion with upstream promoters was performed at UT Austin and allowed to increase both copy numbers and expression levels of the resulting replicative vectors [218], which may possibly give a new impulse to the use of such episomal vectors. Interestingly, the multipurpose pYL15 vector that was used to establish Cell Atlas (cf. Section 3.1.3) by enabling expression of heterologous genes into recombinant fluorescent fusion proteins by addition of a GFP tag, was designed to be used either as a centromeric replicative vector or, following restriction digest, as an integrative one targeting the *leu2* locus of Po1g strain [176].

#### 4.1.2. Integrative Vectors and Cassettes

Since the beginnings of *Y. lipolytica* genetic engineering, integrative vectors constitute the preferred tool for either heterologous protein production or metabolic pathway engineering, thanks to the very high stability of integrated TUs, comparable to that of native genes [10,19]. Integration of complete vectors can be targeted to a precise locus in the genome through linearization in a region of homology that, because of the predominance of NHEJ recombination in *Y. lipolytica*, needs to be large enough (0.5–1 kb on each side) [10,215]. Such regions of homology with the genome that allow the targeting of a vector (or of a TU-bearing DNA fragment generated by restriction digest or PCR) are generally chosen in rDNA coding sequences [158,218], in the 3′ non-coding regions of some genes or in the ORF of selected genes which inactivation could be beneficial for the intended purpose (as proposed notably in the modular Golden Gate toolkit developed at INRA [219]).

A popular alternative is the prior construction of a recipient strain bearing an integrated docking platform, which can be for example a bacterial vector backbone (such as the pBR322 docking platform of Po1g strain [159]—cf. Section 3.1.3) or a yeast-derived sequence naturally absent from this strain, such as the zeta docking platform of JMY1212 or JMY2566 [162,189] (cf. Section 3.1.5). As explained in these previous Sections, the docking platforms of these strains allow the easy targeted integration of unique copies of the corresponding vectors at precisely known neutral loci in the genome, facilitating notably enzymatic screening or TU design comparison. The Po1g strain was constructed for easy integration of the pYLEX1 (aka pINA1269 [159]) expression vector or the pYLSC1 (aka pINA1296 [159]) secretion vector, both included in the YLEX kit (cf. Section 2.3.2). The JMY1212 and JMY2566 strains were designed for efficient integration of any of the numerous available zeta-based vectors [42,43,220,221] or, more precisely, of the TU-bearing transformation cassette that needs to be isolated using restriction digest from these auto-cloning vectors. The zeta-based vectors have been developed at INRA as auto-cloning vectors from which a *URA3*-bearing transformation cassette can be isolated and used to transform any *Y. lipolytica* Ura^−^ strain. These zeta-bordered transformation cassettes can integrate by homologous recombination into the zeta sequences that the strain may bear, for Ylt1-carrying strains (or strains equipped with a zeta platform) or can integrate at random sites in strains devoid of this retrotransposon, thanks to the high level of NHEJ from *Y. lipolytica* [60,220].

Some zeta-based vectors bear a defective version of the *URA3* marker (*ura3d4* allele with truncated promoter) in order to promote an in vivo amplification of the copy number of the integrated transformation cassettes [60,158,220]. Despite being a laborious and rather unreliable process, the construction of multicopy strains using this strategy has been fruitfully applied to the production of numerous heterologous proteins, as extensively reviewed previously [19]. These examples contributed to the success of the zeta-based vectors that remained until recently the most widely used tools for engineering *Y. lipolytica*. As a method for amplifying expression, the defective zeta-based vectors now show their limitations: their transformation efficiency is low (dramatically low if random integration is used) while they require testing a high number of transformants to select a good producer. Some promoter engineering strategies, improved during the last decade [222], are now able to drive better expression increases than the use of defective markers, while being more reliable and easier to perform technically (cf. Section 4.2.1).

Using a random integration strategy also present some important drawbacks, notably a lack of control of the construction and a high risk of deleterious effects of the genomic insertions, which are not desirable for industrial production. A precise comparison of random and targeted integration has been performed recently: following the assembly of a β-carotene metabolic pathway in a zeta-based vector, using a Golden Gate Assembly method, the resulting transformation cassette was either integrated at random into the Po1d strain or targeted to the zeta docking platform of the JMY1212 strain. The randomly obtained transformants exemplified highly variable levels of carotenoid production, while the targeted ones showed more reproducibly high yields [223]. Similarly, some instability problems were observed when constructing strains with randomly integrated multiple copies, a factor that impairs the use of such strains for industrial applications, as reviewed recently [55]. For these reasons, the use of targeted integration at selected genomic loci or docking platforms is now considered as a much more reliable strategy.

#### 4.1.3. Multiple Transcription Unit Vectors, In Vitro DNA Assembly Methods and *Y. lipolytica* Toolboxes

Following the first example of a tandem dual cassette vector, used for engineering Po1g strain for gamma-linolenic acid (GLA) production [224], multiple TUs vectors have been designed in different laboratories. Notably, an integrative vector carrying three TUs was applied to engineering *Y. lipolytica* for improved glycerol metabolism and the resulting strain demonstrated a high stability of the triple integrated expression cassette, despite the presence of tandemly repeated promoter and terminator sequences [225]. The record of TU number and overall vector size belongs to a very large (19 kb) replicative vector carrying 5 TUs that was applied to engineering Po1g strain for violacein biosynthesis [215]. Despite some lack of evidence for estimating the risk of reshuffling problems when using repeated sequences, the research teams who developed toolboxes for *Y. lipolytica* engineering tend to privilege the use of different promoters and terminators as multiple TU elements in order to minimize the possibilities for homologous recombination. Interestingly, a recent process development study using a starch-utilizing GM *Y. lipolytica* for lipid production suggested that, beside TU design itself, the positional order of multiple TUs was an additional factor to be considered for optimized results [226].

The violacein pathway construction was obtained using the YaliBricks system, an in vitro assembly method that allowed the assembly of the 12 kb five-gene violacein biosynthetic pathway in only one week [216]. The YaliBricks system consists in a modular assembly method designed at the University of Maryland (Baltimore County) that complies with the BioBrick standards by using four distinct but ligation-compatible restriction sites for allowing the one-step assembly of several DNA fragments. A library of YaliBricks vectors, carrying various elements for assembly of *Y. lipolytica* TUs (including 12 different promoters) was designed [216], further completed with CRISPR-Cas9 tools [217] (cf. Section 4.3) and all these plasmids are, to the best of our knowledge, intended to be deposited on the Addgene nonprofit repository platform (www.addgene.org (accessed on 3 June 2021)).

A different in vitro assembly method is proposed in the multipurpose EasyCloneYALI toolbox developed at the Novo Nordisk Foundation Center for Biosustainability (Technical University of Denmark): their expression toolkit comprises a series of 26 auto-cloning vectors compatible with USER (uracil-specific excision reaction) cloning, a method employing a USER enzyme to create single-stranded extensions in PCR fragments allowing directional seamless assembly of DNA fragments [227]. These EasyCloneYALI vectors are designed to target the integration of TUs at 11 intergenic sites chosen to allow both an unaffected growth of the recipient cell and a high expression level of the gene of interest; they are available from Addgene [227]. A second toolkit, for CRISPR-Cas9 tools, was also developed (cf. Section 4.3.1).

At last, the Golden Gate Assembly (GGA) method, using Type IIs restriction enzymes (cutting DNA at distance from the recognition site) to generate DNA fragments with variable non-palindromic overhangs that allow their one-step directional assembly, has been chosen at INRA for developing a modular Golden Gate *Y. lipolytica* expression toolkit [219,223]. The resulting toolbox, available from Addgene, comprises an auto-cloning zeta-based destination vector backbone and a series of 64 plasmids, bearing as many different GG biobricks (including nine promoters) for one-step GGA of auto-cloning expression vectors carrying one, two or three TUs. The resulting integration cassettes can be inserted at random in the genome or targeted to four selected loci, a zeta docking platform and three ORFs from genes which inactivation could be beneficial for industrial applications, namely *LIP2*, *GSY1* and *MFE* [219]. This system was applied notably to optimizing expression from a β-carotene biosynthesis pathways using a promoter shuffling approach. Subsequent integration of this optimized GG-assembled pathway into a Po1d-derived obese *Y. lipolytica* strain, in two copies, generated the best β-carotene yield ever obtained from a microbial producer [228]. This GGA platform was also successfully applied to the assembly of a three-gene metabolic pathway allowing xylose utilization by *Y. lipolytica* [219]. More recently, some CRISPR-Cas9 tools were also added to this GGA toolbox (cf. Section 4.3.1). Very recently, the modular and combinatorial properties of the GGA method has been applied to optimizing violacein production in a collaborative work between the University of Maryland and Jiangnan University. These authors constructed a library of violacein producing GM Po1g strains in which each of the five genes from the violacein pathway could be expressed from any of three promoters of different strengths [82]. The GGA efficiency was maximized by selecting the more effective linker sequences. An optimization of the cultivation conditions of the selected best producer, using the strongest promoter for all genes, lead to the unprecedented yield of 70 mg/L of violacein [82]. This work constitutes a proof of concept of how the construction of GG-assembled pathway libraries with randomized promoter strengths could maximize the output yield from any given heterologous (or possibly native) metabolic pathway.

#### 4.1.4. In Vivo DNA Assembly Methods by Homologous Recombination and Artificial Chromosomes

Despite the high level of NHEJ in *Y. lipolytica*, a few research groups have attempted rather recently to use homologous recombination (HR) for in vivo assembly of metabolic pathways in this yeast. A consortium of Shanghai laboratories was able to obtain the assembly and integration at the rDNA locus of a *Y. lipolytica* strain of an 11 kb and three-gene β-carotene synthesis pathway in only one week [229]. The whole process included two steps: first, the separate in vitro assembly of each of the 3 TUs from the new pathway and of the one for the selection marker, by overlap extension PCR and the co-transformation of yeast cells with these four elements that were directionally assembled and integrated, by in vivo HR between their respective overlaps. The overall efficiency of this one-step in vivo assembly/integration process was around 20% despite the use of small overlaps, of only 65 bp, between the four TUs. The external regions of homology with the genome that allowed to target the final integration of the construct to the rDNA locus were however larger, around 0.6 kb [229]. The screening of successfully engineered yeast cells was facilitated by the orange/red color of positive colonies, due to efficient β-carotene biosynthesis. Some of the best producing transformants, with the deepest color, revealed the presence of an additional integration of some elements from the new pathway, indicating a lack of control of the progress probably imputable to NHEJ events [229]. The same strategy, applied to the assembly of a different 10 kb β-carotene synthesis pathway, in either a control strain or an NHEJ-defective one (*ku70*/*ku80* double deletion), demonstrated that the assembly/integration was strongly enhanced when NHEJ was inactivated, with an overall efficiency as high as 63% [230]. A similar strategy of one-step in vivo assembly/integration was applied at Nanjing Tech University for a 10 kb and three genes arachidonic acid (ARA) metabolic pathway [231]. This work assessed the effect of the overlaps’ length on the assembly/integration efficiency, demonstrating an optimum of 23% when overlaps of 1 kb were used. The ARA-engineered Po1f strain selected in this study was also shown to exhibit a high growth rate and a genetic stability compatible with industrial use [231]. The HR efficiency of *Y. lipolytica* can thus be sufficient for driving effective in vivo assembly and integration of complex structures, especially in an NHEJ-deficient genetic background.

These interesting possibilities have been very recently pushed further in an innovative work from Toulouse Biotechnology Institute (TBI, Toulouse University, Toulouse, France) who have been able to obtain the in vivo assembly, in a Po1d derivative, of a 23 kb artificial chromosome [232]. The ylAC plasmid that serves as basis for the whole construct can be digested with different restriction enzymes in order to generate three DNA fragments, each carrying a different selection marker gene, corresponding to the two telomeric ends and to the central region of the future artificial chromosome. These three selectable elements are designed to incorporate, during in vivo assembly, two additional DNA fragments corresponding to one or two new metabolic pathways (TUs). Preliminary experiments have been necessary for choosing promoters for the TUs and for determining the optimal ylAC/TUs ratio for in vivo assembly. These assays have also established that using an essential gene as additional selection marker was mandatory for the long-term stability of the artificial chromosome. An adapted recipient strain, Po1dh, was deleted for 5-aminolevulinate synthase, using CRISPR tools, in order to allow the use of the corresponding *YlHEM1* gene as a selection marker fitted to every cultivation condition [232]. The correct assembly by in vivo HR in Po1dh of the *URA3*-bearing left telomeric arm, of the central region bearing the essential *HEM1* gene, of the *LEU2*-bearing right telomeric arm that also carried an ARS/CEN sequence and of two new metabolic pathways was obtained with a more than 90% efficiency. The two TU DNA fragments were obtained by PCR and designed to present 50 bp bordering homologies with the sequences at the extremities of those of the ylAC restriction fragments they needed to be linked to [232]. The proof of concept of the efficiency of the ylAC system was demonstrated by simultaneously engineering *Y. lipolytica* for xylose utilization and for cellobiose catabolism. In less than a week of wet laboratory experiments, the one-step in vivo assembly of two heterologous metabolic pathways, each composed of three genes, was performed and the resulting 23 kb artificial chromosome exhibited a stability comparable to that of a natural chromosome [232]. This innovative artificial chromosome strategy represents a powerful new tool for both academic and applied research on *Y. lipolytica* and the ylAC plasmid is intended to be deposited at Addgene. This remarkable addition to the global *Y. lipolytica* toolbox is expected to be highly beneficial for the engineering of industrial strains.

### 4.2. Functional Elements for the Design of Expression Cassettes

A wide range of functional genetic elements are available in *Y. lipolytica* for the design of TUs adapted to the peculiar purpose of each engineering purpose. They comprise some regulatory components, promoters and terminators and some optional targeting components that can be fused to the heterologous ORF in order to address the recombinant protein to different organelles or to the vesicular secretion pathway. Secreted proteins can be either released into the cultivation medium or exposed on the surface of the cell (surface display systems). All these functional elements have been extensively described and compared in previous reviews [19,20,45,54] and will only be resumed here. During the early development of *Y. lipolytica* engineering, in the 1980s, the first regulatory and targeting components to be used were those from the few identified genes corresponding to highly secreted enzymes, namely AEP (encoded by the *XPR2* gene) and extracellular LIP2 lipase [18]. The molecular biology progresses now allow the selection of promising candidate genes by mining genome-wide omics data and to fine-tune the properties of native elements through genetic engineering [54].

#### 4.2.1. Natural *Y. lipolytica* Promoters and Promoter Engineering

The choice of a promoter is of paramount importance for the design of any engineering project: for producing a heterologous protein, strength and either constitutivity or inducibility by a process-friendly inducer are required; for adding a new (or optimizing a native) metabolic pathway, some tunability of the expression for each gene is expected. Among natural promoters, the inducible *XPR2* promoter (noted p*XPR2*), used at first for its high strength, presents a complex regulation [233], not compatible with industrial processes. Other notable natural promoters include the strong constitutive p*TEF* [234] and the inducible p*POX2* [235] and p*EYK1* [236]. Together with its improved version obtained through intron-mediated enhancement, p*TEF*in [237], p*TEF* (from the translation elongation factor 1-α gene) still represents a preferred choice for applications that require a strong constitutive promoter. In contrast, known inducible *Y. lipolytica* promoters, mostly derived from lipid pathways (including p*POX2* from an acyl-CoA oxidase gene), have until recently seen their industrial use impaired by incomplete substrate repression and by the hydrophobic nature of their inducers [19,20]. Fortunately, a study from INRA, Liege University and Gembloux Agro-Bio Tech (Belgium) recently showed that p*EYK1* (from an erythrulose kinase gene) was strongly induced by erythritol and erythrulose, thus providing the non-hydrophobic inducer system that was previously lacking for *Y. lipolytica*. These compounds can be used as non-metabolized inducers in a Δ*EYK1* genetic background [236].

Very recently, a research team from Novogy Inc. (Cambridge, MA, USA) has identified four native *Y. lipolytica* promoters able to downregulate the expression of genes at the transition from growth phase to lipid accumulation one [238]. These promoters could be used to restrict to the growth phase the expression of native or heterologous genes which product would be undesirable during the lipid accumulation phase, with no need of medium change during fermentation. This series of new promoters able to drive lipogenesis phase-specific changes in the expression pattern will constitute valuable tools for many projects aiming to optimize lipid yield and composition in *Y. lipolytica* [238].

The construction of recombinant tailored promoters as an improved alternative to natural ones has been initiated since the 1990s at INRA. Based on a functional dissection of p*XPR2* that highlighted the strength and independency from cultivation conditions of its proximal upstream activating sequence (UAS) [173,239], a functional DNA sequence, UAS1B, was selected for the design of a series of recombinant promoters [159]. In particular, the hp4d promoter, composed of 4 tandem copies of UAS1B and a core p*LEU2*, demonstrated remarkable properties for heterologous protein production: in culture media of various composition and pH, hp4d demonstrated a high growth-phase dependent expression profile that increases at the beginning of the stationary phase [240]. This peculiar characteristic naturally allows a partial dissociation of growth and expression phases, maximising productivity and minimising potential toxicity concerns. This recombinant hp4d promoter rapidly became, and still remains, the most used worldwide for heterologous expression, as reviewed previously [19,20,54]. It has been selected for use in the vectors of the Yeastern YLEX commercial kit (cf. Section 2.3.2).

Thanks to the rapid progress of molecular biology technics, the concept of multi-UASs recombinant promoters has been more recently pushed further at UT Austin: these authors generalized the concept of “hpNd” tailored promoters by inserting from 1 to 32 copies of UAS1B (aka UAS1*_XPR2_*) upstream of core promoters from either p*LEU2* or p*TEF*. This new series of recombinant promoters could drive a wide range of expression levels, the strongest ones being as much as eight-fold more powerful than the best natural *Y. lipolytica* promoters [222]. Surprisingly, this study showed that natural yeast promoters could be considered as “enhancer-limited”, and that transcription factor availability did not constitute a limiting factor for expression level. In addition to UAS1*_XPR2_*, other enhancer elements have been made available as building bricks for the design of new recombinant promoters, notably the constitutive UAS*_TEF_* [241], the erythritol/erythrulose-inducible UAS1*_EYK1_* [236] and the fatty acid-inducible UAS*_POX2_* [242]. This latter enhancer element was used in the design of a p*POX2*-derived promoter with an unprecedented 48-fold induction level that could be used not only for metabolic engineering but also as a fatty acid biosensor [242]. New strong inducible recombinant promoters were derived from p*EYK1* by tandem addition of multiple UAS1*_XPR2_* or UAS1*_EYK1_* enhancers [236]. At last, a more recent analysis of p*EYK1* and p*EYD1* (from another erythritol catabolism gene) through phylogenetic footprinting, mutagenesis and hybrid promoter construction using GGA allowed the identification of new enhancers (UAS1_EYD1_ and UAS2_EYD1_) and the design of a series of derived inducible tailored promoters of variable strengths for various applications, from industrial protein production to fine-tuning of gene expression levels in metabolic engineering projects [243]. The tight basal repression level of p*EYD1* allowed the design of derived tailored promoters in which erythritol-based induction could be up to more than 30-fold in a wild-type strain and up to nearly 900-fold in a Δ*EYK1* genetic background [243]. Using the strongest promoter available can however sometime be counterproductive, as was demonstrated in a study comparing recombinant promoters of different strengths, with 2 to 8 UAS1_XPR2_, to produce several secreted proteins of industrial interest: depending on the protein, either the strongest or the weakest promoter produced the best yield, exemplifying how translation and post-translational traffic could constitute limiting steps for some secreted proteins [244]. This study thus suggested that promoter suitability could be protein dependent and that introducing a gene of interest into a pool of vectors carrying different promoters of variable strength can be a powerful strategy to identify the more appropriate one.

In addition to the search for new enhancer elements, a consortium of laboratories from Tianjin University (Tianjin, China) proposed an alternative approach for the improvement of recombinant promoters by engineering the core promoter region [245]. The potential role of this region of 20–80 bp between the TATA-box and the transcriptional starting site may have been underestimated, although some early studies had noticed that it may exert some influence on expression [159]. The authors of the Chinese study used a high-throughput approach to screen a library of random 30 bp DNA sequences for their efficiency as artificial core in recombinant promoters directing the bioconversion of lycopene to β-carotene, as reported by changes in color of *Y. lipolytica* colonies. The sequence of these artificial core promoter was shown to strongly influence the strength of the recombinant promoter, with the presence of T-rich elements and a low GC percentage favoring high expression, a feature also found in strong natural yeast promoters [245]. This work allowed a further optimization of recombinant promoters based on p*EXP1* and p*GPD* and added some selected artificial core promoter sequences to the global *Y. lipolytica* toolbox.

#### 4.2.2. Natural and Synthetic Terminators

Since the 1980s and until recently, terminator sequences obtained from the highly expressed *XPR2* and *LIP2* genes have been used in almost all TU design projects [19,20]. The role that terminators can play in efficient gene expression has long been underestimated. Being generally considered as necessary but rather neutral elements from TUs, these terminating regions did not raised a lot of interest besides the question of a potential risk of recombination due to their repeated use in multiple TUs. For that reason, the recent INRA Golden Gate toolkit for expression in *Y. lipolytica* includes 13 different terminator biobricks for multiple TUs design [219]. However, the influence of terminator sequences on transcription completion and mRNA half-life has been rather recently demonstrated in yeasts [246]. As an alternative to natural terminating sequences, some short synthetic terminators designed for *S. cerevisiae*, able to drive a four-fold increase of transcription and expression in this yeast, were shown to be also functional in *Y. lipolytica* [246]. Additionally, the smaller size of these terminators compared to that of natural sequences (35–70 bp versus 100–430 bp) constitutes an advantage for TU and vector design and both their size and synthetic nature minimize the risk of undesired HR between TUs or with the genome. These short synthetic terminators are therefore expected to ease TU construction and to contribute to a higher stability of heavily GM strains.

#### 4.2.3. Targeting the Secretion Pathway: Secretion Signals and Surface Display Systems

Vesicular secretion of heterologous or recombinant proteins, which notably facilitates their recovery from the cultivation medium, is obtained by N-terminal fusion of secretion signals to the ORF of the mature protein sequence. In *Y. lipolytica*, the pre or prepro regions from either *XPR2* or *LIP2* genes, encoding the major secreted enzymes, have been used for that purpose since the 1980s, together with *XPR2*/*LIP2* prepro hybrid sequences [19,20,42,247,248]. Up to now, the smaller and efficient *XPR2* pre sequence [210] is generally a preferred choice and this secretion signal was selected for the pYLSC1 secretion vector from Yeastern YLEX commercial kit (cf. Section 2.3.2). Alternatively, some heterologous secretion signals have been successfully used in *Y. lipolytica*, especially when from plant or fungal origin, as extensively reviewed previously [19].

More recently, research teams from Poznan University of Life Sciences (Poland) have used genomic and transcriptomic data mining combined to functional screening of a library of GG-built secretion cassettes to identify five new highly efficient secretion signals and to define a consensus sequence for a potentially robust synthetic secretion signal [249]. These new GG-compatible secretion signals constitute valuable additions to the global *Y. lipolytica* molecular toolbox. They were notably used in a synthetic biology study in which a TU and signal peptides shuffling approach allowed the optimization of a starch-utilizing GM *Y. lipolytica* strain for lipid production [225].

The development of surface display methods, for exposing recombinant secreted proteins on the cell surface (arming yeast), is relatively recent in *Y. lipolytica*. Surface display technologies have numerous biomedical and biotechnological applications. Notably, the armed yeasts equipped with various new functionalities can be used as whole-cell microbial factories that can be easily separated from the bioconversion product of interest. The first *Y. lipolytica* surface display method was developed at OUC (China) by C-terminal fusion of the ORF of heterologous genes to a sequence encoding the 110 last amino acids of CWP1, a native cell wall protein [221]. This C-terminal part of *CWP1* gene corresponds to a GPI anchor domain, namely a signal for the posttranslational addition of a GPI (glycosylphosphatidylinositol) structure to the secreted protein and for its covalent linking to β-1,6 glucans from the yeast cell wall (surface display). As reviewed previously [20], a few other GPI anchoring signals from *Y. lipolytica* cell wall proteins have also been applied to surface display in this yeast [250,251,252], but the OUC/INRA zeta-based auto-cloning pINA1317-CWP110 surface display expression vector [221] remains up to now the more widely used tool for constructing arming *Y. lipolytica* cells.

As reported previously [20], a few GPI-independent alternative strategies have also been applied to surface display on *Y. lipolytica* cells, by fusion of the recombinant protein with the flocculation domain from either *Y. lipolytica* or *S. cerevisiae* Flo1 [250,253] or with Protein Internal Repeat (Pir) or Chitin Binding Module (CBM) domains from *Y. lipolytica* cell wall proteins [254,255].

#### 4.2.4. Targeting Organelles for Subcellular Compartment Engineering

Instead of being released in the cytoplasm of the cell, recombinant proteins can be specifically addressed to different subcellular compartments through transcriptional fusion of their ORF with the corresponding targeting sequences. They can be retained in the ER if a C-terminal Lys-Asp-Glu-Leu (KDEL) amino acid sequence is present or targeted to the cell peroxysomes if a C-terminal tripeptidic peroxisome targeting signal (PTS) is added, the amino acid sequences Ala-Lys-Ile (AKI) or Ser-Lys-Leu (SKL) being the more effective ones in *Y. lipolytica* [57,100,256]. Recombinant proteins can also be addressed to LB and oleosomes through transcriptional fusion of their ORF with the C-terminal amphipathic domain of a heterologous oleosin, a structural protein associated with the single layer membrane of plant oil bodies [100,104]. This oleosome targeting strategy was developed at first for the design of tunable functional nanoparticles that could be assembled in *Y. lipolytica* cells for various biotechnological application [104], as described in Section 2.3.5.

In addition to classical intracellular expression, the possibility of targeting the different recombinant enzymes from a remodelled or newly introduced metabolic pathway to the ER, the peroxysomes or the LB of *Y. lipolytica* cells constitutes a new and powerful tool for fine-tuning the metabolic engineering of this yeast. This novel strategy of compartmentalization of the engineered pathways in different cell organelles has been fully exploited in a recent work from the Huazhong University of Science and Technology (Wuhan, China): the simultaneous targeting of an engineered lipase-dependent metabolic pathway to ER, peroxisomes and LB allowed to maximize the yield of biofuels produced in the resulting GM *Y. lipolytica* strains, as evoked in Section 2.3.4 [101]. Such recent possibilities of compartmentalized metabolic engineering will facilitate complex engineering strategies that aim at maximizing the yield of a compound of interest by a holistic approach, taking into account not only the targeted pathway and its possible bottlenecks but also the necessary up-regulation of the cofactor and/or anti-oxidative pathways.

#### 4.2.5. Selection Marker Genes and Marker Rescue Systems

Various marker genes can be used for GM strain selection in *Y. lipolytica*: several genes from amino acids or uracil biosynthesis pathways for auxotrophy complementation, some resistance markers and a few catabolic markers that can be selected by the ability to use a given substrate as sole carbon or nitrogen source [19,20,54]. If auxotrophy markers are the most commonly employed due to their historical incumbency and their simplicity of use, they require the preliminary construction of the corresponding auxotrophic strains, which constitutes a drawback for the valorisation of natural strain biodiversity. In contrast, catabolic and resistance marker genes constitute dominant selection markers that can more easily be applied on a larger scale to the screening of strains for a given application.

Among the available auxotrophy markers, *LEU2* and *URA3* genes have been and remain the most commonly used, according notably to the popularity of the Po1 series of recipient strains bearing *leu2* and/or *ura3* non-leaky non-reverting mutations (cf. Section 3.1.3 and Figure 2). Other less common choices are *LYS5* and *TRP1* genes, as reviewed recently [54]. The availability of *Y. lipolytica* genomic sequences and the new possibilities for easier engineering, such as CRISPR tools (cf. Section 4.3), are expected to facilitate a future increase of the range of auxotrophic markers and corresponding multi-auxotrophic strains. Despite the popularity of *LEU2* as a selection marker, some rather recent studies have raised concern about a possible impact of leucine metabolism on lipogenesis in *Y. lipolytica* [180,257], a factor that pleads in favour of the use of other auxotrophy or preferably dominant markers, at least for lipid-related metabolic engineering projects.

The *URA3* gene constitutes since the 1990s a favorite choice when a marker rescue step is to be used, thanks to its easy counter-selection using the toxic uracil precursor analog 5-FOA (5-Fluoroorotic acid) [258]. Marker rescue consists in restoring an auxotrophy by deleting an integrated selection marker, for allowing its repeated use in multiple sequential engineering steps. More recently, a counter-selection system has also been made available for the *TRP1* gene in *Y. lipolytica*, using the toxic analog 5-fluoroanthranilic acid (5-FAA) [259]. The Cre-lox recombination system from bacteriophage P1 has been used at INRA to design a user-friendly system for gene deletion and marker rescue in *Y. lipolytica*: any integrated loxP-bordered marker TU can be efficiently excised through transient expression of the heterologous Cre recombinase using a replicative vector [215]. Different loxP-excisable markers have been included in some of the newly developed *Y. lipolytica* toolboxes, the two EasyCloneYALI toolkits [226] and the Golden Gate toolkit CRISPR tools [260]. Alternatively, marker rescue can be obtained through a blaster cassette strategy, by making use of the enhanced HR rate of a Δ*Ku70* genetic background to allow the use of short repeated DNA bordering sequences for excising a marker for which a counter-selection is available. Notably, a TRP-blaster system and a URA3-blaster cassette using 100 bp bordering repeats have been described [259,261].

Unexpectedly, some transposomics tools, more generally associated with massive genome studies such as Tn-seq (cf. Section 4.3.4), can also be used to enable scarless precise excision of a selection marker following integration of a TUs-bearing cassette by classical or CRISPR-based methods. Conceived as an alternative application of the in vivo transposition system based on *piggyBac* transposon described below (cf. Section 4.3.4), this tool can constitute an efficient marker rescue system following classical or CRISPR-mediated marker-selected integration of exogenous pathways, by allowing scarless removal of the selection marker, for reiterated use or safety regulation purposes. This alternative use of the *piggyBac* in vivo transposition system makes use of an excision-only transposase variant, heterologously expressed from a replicative vector, to obtain the excision from the genome of any DNA fragment bordered by *piggyBac* inverted terminal repeats (ITRs) [177].

Even though naturally resistant to most common antibiotics, *Y. lipolytica* cells are sensitive to hygromycin B, to nourseothricin, to glycopeptide antibiotics of the bleomycin/phleomycin group and to mycophenolic acid, so that the corresponding *hph* (Hyg^R^), *NAT* (NTC^R^), *ble* (Phleo^R^) and *guaB* genes can be used as dominant markers [177,227,262,263]. The Hyg^R^ gene indeed constitutes a preferred marker in the INRA gene disruption and marker rescue Cre-lox-based system [215]. Together with NTC^R^, this dominant marker was included in the two EasyCloneYALI toolkits [227] and in the Golden Gate global toolkit [219,260].

Interestingly, the *ScSUC2* TU integrated in W29-*ura3-302* and the derived Po1 series of strains (cf. Section 3.1.3 and Figure 2) was intended at first to be used as a catabolic dominant marker when sucrose was used as sole carbon source [15], but was preferably applied to recipient strain improvement through sucrose consumption [10,54]. An optimized version of this invertase TU using the p*TEF* and its native secretion signal [126] was included as dominant marker in the Golden Gate toolkit [219]. A second catabolic dominant marker, using a *DsdA* TU encoding a bacterial D-serine deaminase to allow cell growth on D-serine as sole nitrogen source [264], has been included into the EasyCloneYALI toolbox [227].

These dominant resistance or catabolic markers can be used to engineer directly wild-type strains, which could allow leveraging *Y. lipolytica* natural biodiversity for biotechnological application. Such tools could enable selecting among a large range of wild-type isolates for peculiar characteristics well-adapted to each intended application, an objective pursued by more and more research teams [118,171,260,265]. They could also be seen as potential tools to be assayed for attempting to engineer other yeasts from the *Yarrowia* clade for biotechnological applications, as evoked in Section 2.4.2 [117,118,121].

### 4.3. Gene Editing and Whole Genome Analysis Technologies: CRISPR and Other Tools

Since not more than a decade, a number of innovative genome editing and massive analysis strategies have emerged, based on different bacterial defense systems, notably on the very popular 2020 Nobel-Prize-winning CRISPR system or on some in vivo transposition systems. These New tools have rapidly been adapted for use in numerous organisms, including *Y. lipolytica*, as reviewed previously [54,207,266,267] and resumed below.

#### 4.3.1. CRISPR Tools and *Y. lipolytica* CRISPR-Cas9 Toolboxes for Gene Editing

The numerous CRISPR (clustered regularly interspaced short palindromic repeats) tools that have been derived from the bacterial Cascade (CRISPR-associated complex for antiviral defense) system are all based on the production, into the cell nucleus, of a nuclease (generally Cas9) complexed with a synthetic single guide RNA (sgRNA) that targets it to the homologous genomic locus into which a double-strand break (DSB) needs to be introduced [268]. This DSB will be repaired either by NHEJ, generating insertion/deletion (indel) mutations or, if a donor sequence is present, by homology-directed repair. In presence of a TU(s)-bearing DNA fragment flanked by homologies to the genomic DSB site, this cassette will serve as a repair template so that the TU(s) will be integrated by HR at the targeted locus. Such a powerful CRISPR–Cas9 tool can be used for example to obtain, without the need of a selection marker, the disruption of a target gene and the simultaneous integration of a new metabolic pathway [268]. Adapting CRISPR tools to a new organism can constitutes a challenge, since it necessitates the fine-tuned expression of both Cas9 nuclease and sgRNAs, together with the intranuclear targeting of the heterologous enzyme under a fully functional form. The first *Y. lipolytica* CRISPR-Cas9 systems have been developed independently in 2016 during a collaboration between the University of California at Riverside (Riverside, CA, USA) and Clemson University (Clemson, SC, USA) and by a consortium of laboratories from Shanghai (China) [269,270].

The American research teams combined on a unique pCRISPRyl replicative vector the expression of a codon bias optimized *Cas9* gene from an 8UAS1-pTEF promoter and the expression of the sgRNA of choice from a recombinant promoter, fusion of a native RNA-PolIII promoter with a tRNA, allowing to use endogenous tRNA processing for improved mature sgRNA production. This pCRISPRyl vector, which can be applied to CRISPR-Cas9 markerless gene disruption and/or targeted integration of a HR donor sequence, was deposited at Addgene [269]. The same authors also screened *Y. lipolytica* genome for loci that could integrate heterologous TU with no impact on cell growth and five of the corresponding sequences were included in the CRISPR toolkit, as five pairs of vectors targeting each of these neutral loci (for each selected locus, a pCRISPRyl vector expressing the corresponding sgRNA and a corresponding homology donor vector, carrying a GFP TU as fluorescent tag, all available from Addgene). The five selected loci include the two extracellular protease *XPR2* and *AXP1* genes, which disruption is beneficial for heterologous protein production (cf. Section 3.1.3). A proof of concept of these CRISPR tools was made through rapid integration of four genes from a semisynthetic metabolic pathway for lycopene production, at four of the neutral loci [271]. The co-transformation of pCRISPRyl and of a donor vector demonstrated markerless HR integration of the donor cassette with a 64% efficiency in a Po1f strain and a 100% one in an NHEJ-disrupted Δ*Ku70* derivative [269]. The Shanghai research teams also designed a unique replicative vector to express *Cas9* and the desired sgRNA, both from p*TEF*in promoters (pCAS1yl vector, deposited at Addgene), with the possible addition of a homologous donor DNA cassette on the same plasmid (pCAS2yl vector) [270]. The pCAS1yl vector demonstrated markerless gene disruption (indel knocking-out by NHEJ) with a more than 85% efficiency in a Po1f strain. Simultaneous double or even triple gene disruptions were also demonstrated, using a pCAS1yl vector carrying two or three tandem sgRNA TUs [270].

More recently, the Novo Nordisk Foundation Center for Biosustainability (Denmark) added some CRISPR tools to its multipurpose EasyCloneYALI toolbox (cf. Section 4.1.3): the EasyCloneYALI toolkit for gene editing comprises a series of 15 pCfB vectors for gene deletion and/or markerless TU integration, all available from Addgene [226]. This EasyCloneYALI CRISPR toolkit, includes two integrative vectors for *Cas9* expression from p*TEF*, carrying a loxP-excisable Hyg^R^ or DsdA dominant marker, a replicative vector for expression of the sgRNA of choice from p*Pot1*, carrying a loxP-excisable NTC^R^ dominant marker, six disruption vectors with sgRNAs targeting six selected loci and the six corresponding donor vectors for integration of the TU(s) of choice at these loci. As a proof of concept of the stability of the GM strains obtained with this CRISPR system, as much as 11 steps of successive TU integrations were performed with no detectable loss of the previously integrated TUs [227].

A consortium of French research teams from INRA, INSA Toulouse and Toulouse University also designed a *Y. lipolytica* CRISPR-Cas9 system and applied a holistic approach to studying the NHEJ-based gene knocking-out process, using multiparameter flow cytometry combined to genotypic and phenotypic analyses [272]. These authors demonstrated that the limiting factor during the RNA/protein complex formation was not Cas9 nuclease availability but sgRNA design, sequence and level of expression, while providing a more general insight into the metabolism of small RNAs in yeast cells. The genome of a *Y. lipolytica* knocked-out strain was then fully sequenced, with no evidence of undesired sequence change, which confirmed the safety of CRISPR-Cas9 tools for gene editing in *Y. lipolytica* [272].

Some CRISPR-Cas9 tools compatible with the GGA method (cf. Section 4.1.3) have also been designed recently for *Y. lipolytica*. A consortium of Austrian laboratories developed a GoldenMOCS-Yali toolkit for expression and CRISPR-Cas9-based metabolic engineering in *Y. lipolytica*, explicitly aimed at leveraging natural biodiversity [265]. Their vectors are compatible with previous GoldenMOCS tools, corresponding to a rapid GG cloning strategy used in multiple species. A CRISPR-Cas9 GoldenMOCS-Yali vector was used for knocking-out a gene into different *Y. lipolytica* wild-type strains, with an efficiency ranging from 6% to 25%, depending on the genetic background [265]. Similarly, the stated intention of valorizing the biodiversity of wild-type *Y. lipolytica* strains prompted the design at INRAE of a series of five GGA-compatible CRISPR-Cas9 vectors, derived from the pCRISPRyl vector, with different loxP-excisable selection markers that include the dominant Hyg^R^ and NTC^R^ [260]. These CRISPR tools are notably compatible with the pool of GG bricks described in Section 4.1.3 [219]. As a proof of concept of the possibility for wild-type strain genome editing, the knocking-out a gene was assayed in nine wild-type isolates and was achieved in seven of them, with a high efficiency in four strains and a 100% success rate in two [260]. These two new sets of CRISPR tools, both available from Addgene, are expected to allow leveraging *Y. lipolytica* natural biodiversity by allowing to select the more appropriate wild-type isolate for each intended application and then to engineer rapidly and easily its metabolism.

However, the low rate of HR in *Y. lipolytica* limits the capacity of this yeast for homology-mediated DNA repair during site-specific CRISPR gene editing. This drawback has been addressed by a MIT research team who improved the CRISPR/Cas9-based methods using tRNA processing for sgRNA production (pCRISPRyl and derivatives) by redesigning the tRNA-sgRNA fusion system through the use of secondary RNA structure prediction. This strategy for improved sgRNA expression resulted in a higher efficiency of CRISPR/cas9 gene editing at chromosomal loci for which it failed or was ineffective when using previous methods [273].

In addition to Cas9, Cpf1 (aka Cas12a) is another nuclease that can be applied to designing CRISPR tools. In contrast to Cas9, which uses naturally two short CRISPR RNA instead of an artificially designed sgRNA, Cpf1 is naturally a single RNA-guided endonuclease. Cpf1 recognizes a less frequent T-rich PAM (protospacer-adjacent motif), instead of a G-rich one for Cas9, and generates sticky ends DSB more distal to the recognition site. These characteristics are expected to allow a more reliably targeted, and possibly repeated, genome editing, which prompted the design of a CRISPR-Cpf1 system for *Y. lipolytica* editing at the University of Maryland, Baltimore County (USA) [274]. This CRISPR-Cpf1 system allowed to perform indel NHEJ-based gene knocking-out with an efficiency in the range of 95% and multiplex editing with a mean efficiency of 79% for double targets and 42% for triple ones [274].

Whatever the CRISPR tool used, the choice of sgRNA sequences remains a crucial step for successful gene editing (or transcriptional control). A study of the relative efficiencies of six candidate sgRNA sequences for each *Y. lipolytica* gene has recently been performed by a consortium of American laboratories, who constructed a library of sgRNAs targeting the 300 first nucleotides of 7854 ORFs from Po1f strain and measured their individual DSB-promoting efficiency in a CRISPR-Cas9 editing assay [275]. This study revealed that 48% of the sgRNA from the six-fold coverage library exhibited a high efficiency and that 95% of *Y. lipolytica* ORFs could be targeted by at least one very highly efficient sgRNA [275]. As reported in more details in Section 4.3.4, this work also provided an innovative multipurpose whole genome analysis method for *Y. lipolytica*.

#### 4.3.2. TALEN Tools for Gene Editing in *Y. lipolytica*

In addition to CRISPR tools, some alternative genome editing strategies are available and a few have been adapted for use in *Y. lipolytica*. TALEN (transcription activator-like effector nucleases) are recombinant restriction enzymes designed by fusing a nuclease with a TAL effector DNA-binding domain that can be engineered to target specific DNA sequences [276]. TALEN are used to generate, at specific genomic loci, DSB that can be repaired through NHEJ or HR in presence of a homologous donor sequence, such as for CRISPR tools. However, for TALEN, the DNA recognition system is part of the recombinant enzyme when, for CRISPR tools, it is brought by the sgRNA. Therefore, the design of a new TALEN for each targeted locus is laborious and costly compared to the more rapid and cheap synthesis of a small RNA. Despite this drawback, a TALEN tool has been used at INSA (Toulouse University) for structure-based mutagenesis of the ketoacyl synthase domain from the *Y. lipolytica* multifunctional fatty acid synthase (FAS), a key enzyme of lipid synthesis pathways, to allow the production of fatty acids with shorter chain lengths [277]. A step-by-step methodological guide of how-to-use TALEN in *Y. lipolytica* for knocking-out or introduction of point mutations into genes was very recently made available by the same authors [278].

#### 4.3.3. Other CRISPR Tools for Base Editing and For Gene Repression or Activation

The targeting ability of the CRISPR-Cas9 system has been exploited in different derived methods in order to allow recruiting new functionalities at a precise genomic locus. The construction of a recombinant Cas9 nuclease fused with an activation-induced cytidine deaminase (Target-AID) has been applied at first for targeting point mutagenesis at precise loci from plant genomes [279]. This innovative method for targeted base editing has been adapted recently to *Y. lipolytica*, at the Seoul National University (South Korea) and applied to multiple gene disruption [280]. The mechanism of this *Y. lipolytica* Target-AID base editor is to use the Cas9/sgRNA complex to recruit a cytidine deaminase (CDA) able to provoke a C to T mutation at the chosen locus, with the aim to create a stop codon within the targeted ORF. In order to obtain (multiplex) gene disruption in a Po1g *ku70Δ::loxP* strain, the sgRNA(s) and a recombinant protein, corresponding to Cas9 fused to both a heterologous CDA and a uracil DNA glycosylase inhibitor, were simultaneously expressed from a pCRISPRyl-derived vector [280]. Using a Δ*Ku70* genetic background is required in order to increase the accuracy of the mutagenesis process by preventing the formation of indels following the cytidine deamination step. The optimization of the fusion enzyme expression level of this Target-AID system enabled performing single gene disruptions with a 94% efficiency and double gene ones with a 31% efficiency [280].

Several CRISPR tools for gene repression or activation have also been designed, which make use of defective versions of Cas9 (dCas9) or Cpf1 (dCpf1) lacking endonuclease activity while retaining their sgRNA-directed DNA targeting function. The University of California, Riverside (UCR) applied their *Y. lipolytica* CRISPR tools (pCRISPRyl replicative vector, cf. Section 4.3.1) to gene repression through the CRISPR interference (CRISPRi) system [281]. At first, they simply used dCas9 to sterically repress gene transcription from sgRNA-targeted loci, with a proof of concept demonstrated by obtaining NHEJ-deficient *Y. lipolytica* strains by directing dCas9 to both *Ku70* and *Ku80* promoters, using multiplex sgRNAs [281]. They were then able to improve the efficiency of gene repression by using a fusion protein corresponding to dCas9 linked to the transcription repressor Mxi1 (Schwartz et al., 2017b). The corresponding CRISPRi vectors (pCRISPRi_Mxi1_yl), empty or bearing the *Ku70* and *Ku80*-directed sgRNAs for NHEJ inactivation, are available from Addgene [281]. A similar CRISPRi system for gene repression in *Y. lipolytica* was developed at Tianjin University, which uses either dCas9 or dCpf1, each alone or fused with a KRAB repressor [282]. This new CRISPRi system uses a one-step GGA strategy and takes advantage of multiplex sgRNA targeting to repress simultaneously the transcription of multiple genes, or to repress more effectively that from a single gene by aiming at several promoter sites. Namely, a high level of repression was achieved (85% when using dCpf1 and 92% with dCas9) when using three sgRNAs to target different sites in the promoter of an integrated *gfp* reporter gene, a strategy that allows avoiding the preliminary screening step usually necessary to select the more effective sgRNA sequence [282]. These CRISPRi tools constitute very valuable additions to the global CRISPR toolbox, as efficient ways to transiently repress any *Y. lipolytica* gene activity and will find a preferred application for temporary repression of NHEJ activity prior to some major genetic engineering step requiring a high HR rate. They will in that case allow avoiding a permanent deletion of *ku70*/*ku80* genes that could impair the robustness of the resulting GM strain.

At last, the CRISPR toolbox from UCR has also been completed with some CRISPR activation (CRISPRa) tools, in a collaborative work with UT Austin. Similar as in the case of CRISPRi, CRISPRa is based on the fusion of a defective nuclease (dCas9) with a transcriptional effector (activator) and makes used of a sgRNA to direct the new (transcription enhancing) functionality to the targeted gene. The synthetic tripartite activator VPR was selected among several candidate activators and, when fused to dCas9, enabled to enhance the transcription of two dormant native β-glucosidase genes, conferring to the GM strain the ability to growth on cellobiose as sole carbon source [283]. The corresponding pCRISPRa_VPR_yl vector is available from Addgene. This new CRISPRa tool pushes further the versatility of the CRISPR-Cas9 system by providing an easy and elegant way to investigate the potential of transcriptionally silent parts from the *Y. lipolytica* genome. As already demonstrated in previous studies for the pentose pathway [71,72] (cf. Section 2.3.3) and confirmed here for cellobiose, mining the *Y. lipolytica* genome for dormant metabolic pathways is a promising strategy for rewiring its metabolism for alternative substrates, and the CRISPRa tools could greatly facilitate this approach. The combined use of CRISPRi and CRISPRa systems is expected to enable fine-tuning *Y. lipolytica* metabolic pathways and to ease complex engineering project. A methodological guide of how-to-use these new tools was very recently made available [284].

More generally, UT Austin also proposed a methodological guide for using the whole series of CRISPR-Cas9 vectors they developed (or contributed to develop) for either genome editing or dCas9-based transcriptional control [285].

#### 4.3.4. Transposomics and CRISPR-Derived Tools for Whole Genome Analysis in *Y. lipolytica*

The development of whole genome analysis tools applicable to functional genomics studies is rather recent in *Y. lipolytica*, despite their interest for holistic approaches of metabolic engineering strategies. The basic idea of such tools is to obtain a genome-wide library of viable mutant cells through a tagging method that allows subsequent massive sequencing of the junction sequences and identification of the corresponding mutated genes. The comparison of the results obtained following growth of the mutant library in various conditions allows to obtain an “inverted image” of the genes contributing to a function of interest. Namely, the surviving tagged mutants can be identified as non-essential and having therefore a low fitness coefficient for the tested growth condition. A preferred whole genome analysis method is transposon insertion sequencing (Tn-seq), which apply transposomics tools to obtaining genome-wide transposon-generated insertional mutants’ libraries and generates fitness coefficients through massive sequencing of transposon insertion junctions.

The first transposomics tools applicable to whole genome analysis have been developed only in 2018 for *Y. lipolytica*, based on a *piggyBac* transposon at UT Austin [177] and on a Hermes transposon (from the dipter *Musca domestica*) at the University of California, Irvine (UCI, USA) [286]. Such tools are based on the mobilization of a modified transposon, corresponding to a selection marker bordered by the transposon ITRs, using a heterologously expressed dedicated transposase (generally adapted to *Y. lipolytica* codon bias and born on a replicative vector). As the *piggyBac* transposition system targets specifically TTAA sequences that are present in less than 2/3 of *Y. lipolytica* ORFs and is also biased in favor of transcribed regions, it presents only a limited interest for whole genome saturation mutagenesis applications such as Tn-seq [177]. This transposomics tool is consequently preferably proposed as a cargo-mobilizing system for marker rescue, as described above in Section 4.2.5. For developing Tn-seq applications, a transposon that integrates as randomly as possible into genomes is required in order to maximize genome coverage. However, the final success of a Tn-seq system will depend on the effective expression of a functional heterologous transposase, a step that can be challenging.

The UCI team have used the Hermes transposon for performing genome-wide saturation mutagenesis into W29 strain. They constructed a library of more than 500,000 insertion mutants that was cultivated for 80 generations on either glucose or glycerol as sole carbon source, before being submitted to massive sequencing in order to identify the mutants present in the surviving population. A statistical analysis of the resulting data identified 22% of *Y. lipolytica* genes as essential and allowed to measure the contributions of non-essential genes to growth on each carbon source [286]. These data were used to evaluate and sometimes correct two previously established genome-scale models of *Y. lipolytica* metabolism [287,288]. A selection of the insertion mutants in which lipid accumulation was enhanced was performed by combining Bodipy lipid staining and fluorescence-activated cell sorting (FACS). The identification of the corresponding genes provided new insights into *Y. lipolytica* lipid metabolism and new targets for future metabolic engineering [286]. The vectors for in vivo Hermes transposition used in this Tn-seq study are available from Addgene. They include a replicative vector carrying both the Hermes transposase TU and the modified transposon (ITR-bordered *LEU2* marker), a markerless version (for inclusion of any marker TU) and some negative control vectors. Depending on the growth conditions compared, this Tn-seq system can be applied to various whole genome studies of *Y. lipolytica* strains for dissection of their metabolism or evaluation of their robustness for biotechnological applications.

In addition to transposomics tools, other methodologies can be applied to whole genome analysis, as long as they allow building a large library of tagged mutant strains. Somewhat surprisingly, the precisely targeted CRISPR tools for gene inactivation can be used for such studies, providing the preliminary construction of a sgRNA library covering the whole genome. This strategy has been chosen by a consortium of American laboratories who were also able to quantify the cutting efficiency of each sgRNA and thus to identify the best sgRNA for each *Y. lipolytica* gene, as described in Section 4.3.1 [275]. The six-fold coverage sgRNA library constructed by these authors was used to transform in parallel three strains with the same Po1f background: a control and two *Cas9*-expressing derivatives, with or without NHEJ disruption (Δ*Ku70*). In presence of a functional NHEJ system, the DSB generated could lead to indel formation, allowing to construct a library of viable mutant cells and to determine a fitness coefficient for each gene. In contrast, the DSB cannot be repaired in the Δ*Ku70* strain, no donor HR sequence being present, which leads to cell death and thus to the obtention of a cutting score for each sgRNA sequence [275]. By comparison of the progeny obtained in presence or absence of native DNA repair, this work provided a high-throughput measurement of each sgRNA-directed cutting efficiency and allowed identifying highly efficient sgRNA sequences for nearly 95% of *Y. lipolytica* genes (cf. Section 4.3.1). This study also improved the identification of *Y. lipolytica* essential genes by a significant reduction of false negatives thanks to the sgRNA redundancy. In addition, the library of viable mutants obtained in *Cas9*-expressing Po1f strain can be used to determine the fitness score of each gene under various cultivation conditions. This CRISPR library was notably applied to the study of lipid metabolism, through the use of a fluorescent lipid dye and of a FACS high-throughput screening system and allowed identifying new target genes which disruption favors lipid accumulation [275].

### 4.4. How Gene Editing Can Leverage Strain Biodiversity and Be a Source of New Engineering Strategies

A few decades ago, laboratories working on *Y. lipolytica* engineering tended to focus on a selected strain and to invest a lot of work and time into introducing one, two or three auxotrophies in its genome in order to be able to use the corresponding genes as markers for further engineering. The first step was generally to obtain an Ura^−^ derivative, thanks to the easy counter-selection of uracil-deficient strains with 5-FOA. The more recent alternative use of dominant markers remained limited and the implementation of marker rescue systems was required for complex engineering projects. In contrast to the more recently developed scarless methods (*piggyBac*, marker-blasters) [177,259,261], these former marker rescue technics presented the drawback of leaving some genetic manipulation vestiges in the genome. Moreover, nowadays, the availability of markerless CRISPR-based genome editing tools [227,260,265,269,270] makes possible the rapid engineering of numerous strains for testing a desired application. As discussed in Section 4.3.1, these new tools can make the valorization of *Y. lipolytica* biodiversity an attainable goal and some of these CRISPR-Cas9 tools, compatible with rapid assembly methods, have been explicitly designed for this purpose [260,265]. These approaches could benefit from the large range of wild-type *Y. lipolytica* isolates made available for research purposes by the yeast collections listed in Table 2. The promising demonstration that some of the tools developed for *Y. lipolytica* could be successfully used to engineer *Y. phangngensis* [122], is also a good omen for the future possibility of extending their usage to other yeasts of the *Yarrowia* clade. In addition to this direct impact of CRISPR-based and other recently developed tools for markerless genome editing, they can also be a source of new engineering strategies by allowing or easing some complex genomic changes, notably for NHEJ disruption or for mating type switching.

#### 4.4.1. Increasing the Homologous Recombination Efficiency in *Y. lipolytica*

As already evoked several times above in this review, *Y. lipolytica* uses predominantly its NHEJ system for the repair of DSB, so that the flanking regions of homology with the genome of a DNA cassette need to be large enough (0.5–1 kb) to allow a correctly targeted integration [10,215]. We have also seen several examples of how this drawback could be palliated by knocking-out *Ku70* and/or *Ku80* gene(s) [230,259,261,269,275,280]. This strategy dates from 2013, when these gene deletions (performed using classical molecular biology methods) have been used to allow facilitating ulterior genetic engineering of Po1d and H222 strains [289,290]. A series of Δ*Ku70* strains derived from Po1g, with different auxotrophies, have also been included in a *Y. lipolytica* toolbox [176]. However, all these NHEJ-disrupted strains exhibit a reduced transformation efficiency and the permanent knocking-out reduces their robustness, as reviewed previously [55].

Some CRISPR-derived tools can now offer an alternative strategy of transient NHEJ repression that allows the combination of the ease of genetic engineering with the possible reversion to a more robust NHEJ-prone phenotype when needed. Namely, the CRISPRi tools described in Section 4.3.3 could find a preferred application in the transient repression of *Ku70* and/or *Ku80* genes, in order to reduce *Y. lipolytica* NHEJ activity prior to genetic engineering, without the drawbacks linked to a permanent knocking-out [281,282].

At last, an alternative approach for increasing homologous recombination in *Y. lipolytica* has been recently proposed by a consortium of Chinese and British laboratories: these authors expressed in Po1f strain a codon-adapted *RAD52* gene from *S. cerevisiae*, encoding the main recombinase from this HR-prone yeast [291]. The heterologously expressed *ScRAD52* improved HR efficiency in *Y. lipolytica* Po1f strain more efficiently than a Δ*ku70* knock-out. The effect was maximized in a Po1f-Δ*ku70*-*ScRAD52* strain, in which the HR efficiency reached 95% when using homology arm lengths of 1 kb [291]. It would be interesting to combine *ScRAD52* heterologous expression with *Ku70*/*Ku80* CRISPRi-mediated transient inactivation to possibly keep the best of these HR enhancement strategies.

#### 4.4.2. Diploid Strain Formation and Sexual Hybridization Following Mating Type Switching

As evoked above in Section 2.2.2 and Section 3.1.2, the frequencies of mating between Mat-compatible wild-type isolates and of hybrid sporulation are very low, probably due to genomic differences linked to the presence of retrotransposons and other retro-elements. The formation of diploid strains would however present several advantages for the design of cell factories, since they exhibit a more robust growth than haploid ones and they can combine the interesting properties from different GM parent strains. Therefore, some early mating-type switching experiments had been reported, as a mean to obtain diploid strains by deleting *MATA* and inserting *MATB* through classical molecular biology methods, in order to obtain the sexual hybridization of a mating-type switched strain with its parental type [292]. If these first assays demonstrated the possibility of obtaining a functional "trans-sexual" strain, the observed mating frequency remained however very low and there was no other report of mating-type switching in *Y. lipolytica* during the following decade. Thanks to the development of new scarless gene editing technologies, this problem of mating type switching has recently been overcome, independently, by a research team at UT Austin and by a consortium of South Korean laboratories, who obtained the efficient formation of diploids between GM strains issued from a same genetic background [74,293].

As evoked above in Section 2.3.3, the UT Austin research team constructed xylose-utilizing strains (XUS) from PO1f and E26 (an obese derivative) strains. They were able to switch the mating type of the E26-XUS strain from A to B, by scarless site-specific HR at the mating type locus using the *piggyBac* cargo-mobilizing system for marker rescue (cf. Section 4.2.5). The crossing of this mating-type switched strain with each of three E26-based type A production strains, genetically engineered to produce, respectively, α-linolenic acid (ALA), riboflavin or triacetic acid lactone (TAL), allowed generating diploid strains producing these new metabolites from xylose as sole carbon source, with yields similar or higher than those of each parental strain from glucose [74]. This sexual hybridization approach could thus allow an easy merging of the properties of two GM strains, which could highly facilitate complex metabolic pathway engineering. The diploid production strain obtained also showed a robust growth and a high stability, demonstrating the high potential of this strategy for the development of efficient cell factories. The satisfactory level of mating frequency observed in these experiments plead in favour of a former detrimentous effect of vestiges that classical genetic manipulations could leave in the genome, a factor that was this time avoided through the use of a more recent scarless gene editing method.

Similarly, the South Korean research teams obtained a mating type switch from A to B in Y2–1U, a GM strain derived from Po1g by deletion of *ku70* and of the six *ACO1-6* β-oxidation genes [293]. The mating type switching was obtained by scarless site-specific HR at the mating type locus using an *URA3*-blaster marker rescue system in a Δ*Ku70* genetic background. The self-mating of this GM strain devoted to the production of dodecanedioic acid, a monomer for polyester and polyamide, was aimed at augmenting its ploidy for obtaining a more robust and efficient cell factory [293]. This second example confirmed that the innovative strategy of diploid formation through mating type switching could ease pathway engineering and bioprocess development. Increasing strain ploidy is indeed expected to enhance genetic stability, stress resistance and productivity of cell factories [293].

### 4.5. Towards a Holistic View of Cell Factories and Bioprocesses Development

#### 4.5.1. The *Y. lipolytica* Pan-Genome

To the best of our knowledge, the sequences of a total of 22 *Y. lipolytica* strains have been published up to now (some others being not publicly accessible), namely, chronologically, those of E150, Po1f, W29, WSH-Z06 and five KGA hyper-producer mutants, A-101, a *ku70* mutant of Po1g, B9, YB-392, YB-419, YB-420, YB-566, YB-567, H222, YlCW001 (ionic liquid-resistant laboratory-evolved derivative of Po1f) and 3 A-101-derived mutants [33,34,36,37,48,125,128,156,160,161,168,171,176]. As some of these strains share a common genetic background (W29 with its derivatives Po1f and g and their mutants; WSH-Z06 and A-101 with their respective mutants), this represents only 10 independent genomes but can however constitute the start of a pan-genome, representative of *Y. lipolytica* genetic diversity. In addition to the usual bio-informatics tools for genomics analysis, the study of eukaryotic microbial pan-genomes requires specific data pipelines, such as the Pangloss tool recently developed at Maynooth University (Co. Kildare, Ireland). This software pipeline can be used to construct a pan-genome from a set of genomes from any eukaryotic microbial species, using a PanOCT (Pan-genome Ortholog Clustering Tool) approach and be applied to various functional characterisation and visualisation analyses of this pan-genomic data. These authors used Pangloss to design a *Y. lipolytica* pan-genome based on a set of seven sequences selected from wild-type isolates of various origins [294]. The *Y. lipolytica* pan-genome still contains a large majority of ORFs which function remains unknown or only putative (http://gryc.inra.fr/ (accessed on 3 June 2021)). The recent development in *Y. lipolytica* of genome-wide high-throughput analysis technics, such as Tn-seq or CRISPR-based ones (CRISPR-directed knocking-out, interference or activation) is expected to allow reducing this number in a near future. As shown above in Section 2.3.3, Section 3.1.4, Section 3.3.3 and Section 4.3.3 for, respectively, pentose, galactose, lactose and cellobiose [48,71,72,187,283], mining the *Y. lipolytica* pan-genome for dormant (or inefficient) metabolic pathways could also allow the improvement of the consumption of alternative substrates, notably through the use of CRISPRa tools.

#### 4.5.2. Genome-Scale Omics Tools and Metabolic Models

As several examples have been given throughout this review and as is tentatively schematized above in Figure 3, new tools and new strategies for metabolic engineering can now result in the envisioning of more ambitious metabolic engineering projects, by implementing efficient in vitro and in vivo assembly methods as well as a wide range of gene editing and regulating tools, notably the CRISPR-derived ones (cf. Section 4.1.3, Section 4.1.4 and Section 4.3). Metabolic engineering projects are now expected to take into account the whole cellular metabolism, including the intracellular metabolic fluxes, the availability of cofactors and the effect of oxidative compounds, and to use the possibility to compartmentalize the metabolic modifications in the different cellular organelles (cf. Section 4.2.4). Nevertheless, any cell factory design relies at first on a precise understanding of cellular metabolism that can only be built through genomic, transcriptomic, metabolomic or fluxomic analyses [295,296,297,298]. Transcriptomics tools have notably been applied, at the Poznan University of Life Sciences (Poznan, Poland), to studying the consequences of an overproduction of secreted heterologous proteins, which can be a metabolic burden and a source of stress for yeast cells [299]. This study established the transcriptomic profile of *Y. lipolytica* when challenged with high-level expression of genes encoding different types of recombinant secretory proteins (small, high cysteine-bearing, highly glycosylated). It identified not only the expected target genes, linked to oxidative and unfolded protein stress, to glycosylation, to folding and to translocation, but also some unexpected ones, linked to non-conventional protein secretion, transcriptional regulation, vacuolar proteolysis or growth arrest. The authors notably hypothesize that *Y. lipolytica* cells could enter into a G1 growth arrest phase during high overproduction of recombinant secretory proteins, in order to withstand this high metabolic burden [299].

White biotechnologies have thus benefited from the rise of multi-omics technologies that allowed obtaining in silico models of genome-scale metabolic pathways, giving a more holistic point of view on cell factory design. Bioinformatics and applied mathematics have thus their role to play, through the design of GEMs (genome-scale metabolic models) in the deciphering of *Y. lipolytica* regulatory networks [287,288,300,301,302,303]. Some more recently developed genome-wide high-throughput tools, such as the Tn-seq and CRISPR-based approaches described in Section 4.3.4 will increase our understanding of cellular processes and allow the verification of the predictions of the GEMs [275,286], a first step towards an improved second generation of such models. In one of the most recent of these GEM studies, a consortium of French laboratories, combined a network interrogation process with a validation step through bench experimentation in order to identify regulatory elements and mechanisms promoting lipid accumulation in *Y. lipolytica* [302]. This study allowed the design of YL-GRN-1, a gene regulatory network comprising more than 100 transcription factors, 4000 target genes and 17,000 regulatory links. Nine new potential regulators of lipid accumulation were identified, from which six were validated in subsequent wet laboratory experiments [302].

Such holistic approaches will probably become much more frequent in a near future, with the data from multi-omics technologies from GM strains being used for in silico modelling of genome-scale metabolic pathways that could identify limiting factors and bottlenecks, providing a second generation of targets for genetic engineering, in a virtuous circle. These systems-level approaches, implemented by high throughput omics data and mathematical modelling, are expected to provide an in-depth understanding of cellular processes for a more rational design of cell factories. The current status of systems metabolic engineering research in *Y. lipolytica* has been the subject of a very recent extensive review (presently online ahead of print) [304].

#### 4.5.3. Adaptative Evolution Strategies and Bioprocess Engineering

Following the choice of a strain and of a metabolic engineering strategy, the next steps of process development are also of major importance in the design of a successful application. They could include some optional adaptative laboratory evolution strategies, which use iterative cultivation steps under a given stress condition to obtain variants better adapted for industrial production, and the bioprocess engineering steps themselves (optimization of medium, fermentation, recovery, purification, etc.).

The use of evolutionary approaches in strain engineering requires the implementation of a suitable selection method for the product and/or phenotype of interest. The FACS methodology constitutes a preferred choice for such purposes. In one of the first examples of using adaptative laboratory evolution in *Y. lipolytica*, for riboflavin overproduction, some research teams from UT Austin and the University of California, San Francisco, compared two FACS methods for high-throughput screening: the classical single cell FACS and the more recent microdroplet-enabled FACS (droplet FACS). It appeared that single cell FACS was favoring intracellular riboflavin accumulation when droplet FACS was favoring extracellular product accumulation [305]. In addition to this example, adaptative laboratory evolution strategies have recently been applied in *Y. lipolytica* for various purposes: restoring the glucose metabolism of a Po1f derivative engineered for succinic acid production [306]; enhancing lipid storage from ACA-DC 50109 strain [135]; selecting the ionic liquid-tolerant YlCW001 strain [161]; improving limonene tolerance during its production by GM strains [307]; enhancing thermotolerance during industrial fermentation for erythritol production [308]; increasing tolerance to aromatic aldehydes or to ferulic acid, both for a more efficient lignocellulose valorization [309,310].

Bioprocess engineering steps will not be detailed in this review, since they have been addressed more appropriately in several previous publications who highlighted, from different examples, the importance of various external factors (temperature, pH, nutrients and oxygen availability) on *Y. lipolytica* growth and productivity, together with the stress that industrial conditions can exert on yeast cells [79,209,311,312,313]. As seen in Section 2.2, this yeast benefits from a robust tolerance to a large pH range, to high salt levels and to organic solvents, all valuable assets in easing bioprocess optimization. Bioprocess engineering progresses have particularly been reviewed for heterologous proteins [208] and biodiesel production [311]. The process optimization implemented for the production of EPA-rich SCO by Dupont (cf. Section 2.3.2) has also been extensively described [58]. In a sustainable development and circular bio-economy approach, a research consortium (Agricultural University of Athens, Greece; City University of Hong Kong, China; IMECAL SA, Spain) has very recently developed a bioprocess using an engineered and evolved Po1f derivative for succinic acid production from zero cost municipal organic bio-waste. The sustainability and commercial viability of this bioprocess were evaluated via techno-economic and life cycle analysis [314].

In the case of a dimorphic yeast such as *Y. lipolytica*, the control of the dimorphic transition is an additional constraint since the cell morphology has a high impact on the production of metabolites and proteins, as reviewed previously [14,65]. The oleaginous nature of *Y. lipolytica* presents major advantages for the bioconversion of hydrophobic substrates since the process can be performed in two-liquid biphasic systems. Thanks to its specific characteristics (naturally secreted emulsifiers, protrusions and cell surface hydrophobicity—cf. Section 2.2.5) allowing an efficient uptake of these substrates from organic solvents, GM strains expressing P450s and their reductases have been applied to the oxidation of hardly soluble hydrophobic steroids in a two-liquid biphasic system. Employing such a biphasic system allowed a more efficient bioconversion compared to an aqueous system while considerably simplifying the whole process [312]. In addition to their interest for the design of metabolic engineering strategies, GEMs can also be applied to predicting the metabolic responses of yeast cells to environmental conditions such as industrial-scale production, as demonstrated at the University of Graz (Austria) during the optimization of a *Y. lipolytica* bioprocess for lipid production [313]. Some statistical modeling tools, such as response surface methodology, are also now currently applied to bioprocess optimization, from wild-type as well as for GM strains [315,316,317,318]. As seen above in Section 2.3.3, a lot of research is performed on remodelling the hydrolytic secretome of *Y. lipolytica* in order to allow the use of plant biomass as a renewable and cheap carbon source, an approach aiming towards sustainable development and circular bioeconomy. The final purpose is to engineer this oleaginous yeast, which natural isolates have only a limited capacity to grow on plant-derived biomass, into a valuable candidate for consolidated bioprocessing applications. Consolidated bioprocessing (CBP) requires the simultaneous production of hydrolases able to degrade plant-derived polymeric substrates and of enzymes allowing the microbial conversion of the released sugars into value-added compounds, in a single step. The progresses that have been made in this competitive research area have been reviewed in a very recent publication, in which the authors discuss the economic advantages of CBP, simulate different industrial CBP models based on GM *Y. lipolytica* and calculate the associated costs [319]. All these tools and strategies will contribute to establish *Y. lipolytica* as a workhorse for a wide range of applications in the very competitive world of white biotechnology.

## 5. Conclusions in the Shape of a Question Mark: What Future for GMOs in Our Societies?

Since the holding of the Asilomar Conference on Recombinant DNA, in 1975 in California (Pacific Grove, CA, USA), during which 140 biologists, physicians or law professionals discussed the potential biohazards and the need for a regulation in the nascent domain of biotechnologies, GMO have been regulated worldwide by governmental agencies [320]. This regulatory framework encompass research on GMOs, which needs to be approved by devoted committees from universities or research institutions, as well as their commercial and industrial use, including that of GMO crops and GMO-containing food. There are however some differences in the regulation between countries, especially concerning the release of GMOs, the most important ones being between the USA and European countries: the American policy is not giving as much attention to the process as other countries and takes into account a concept of substantial equivalence, when the European policy is more severe and holds to the precautionary principle [321]. Notably, the question to know if gene edited organisms (sometimes called new GMOs, including notably CRISPR-generated ones) should be concerned by the GMO regulation received different responses depending on the countries. It was a “no” for the USA, considering that no heterologous sequence was present in the resulting organism (as in the case of a mutagenesis step), but a “yes” for European countries who considered that all organisms generated through genetic engineering are indeed GMOs [322,323]. However, this crucial question still remains presently a matter of societal debate. There are also important differences between social acceptances of GMOs in USA and in Europe, with the Europeans being notably more suspicious towards GM food and considering it more negatively than the North Americans [321]. This situation of strict regulation, especially in Europe, which contributes by the way to reinforce the social reluctance towards GMOs, constitutes a major problem for the development of GMO-based bioprocesses, above all for food applications. These problems have been highlighted in some publications that qualify the classification of GMOs of “meaningless” and this denomination of “nonsensical pseudo-category”, while denouncing the “precautionary rabbit hole” of their regulation [324,325]. If the problems of societal acceptance are particularly acute for GMO crops, this unfavourable situation also impacts microbial biotechnologies. Namely, some agro-industrial or commercial food companies may be brought to revise their strategic research choices, by favouring the use of traditionally improved strains over that of GM ones, despite the fact that these traditional technics may be less efficient and less reliable (and sometimes dangerous for the manipulator, if chemical mutagens are to be used).

To illustrate this point, the case of erythritol is interesting: its industrial production from *Y. lipolytica* by Baolingbao Biology Co. (cf. Section 2.3.1) makes use only of traditionally improved strains and the research papers that describe improvements in the domain also adopted the same strategy. Namely, as described above in Section 3.3.3, a consortium of Chinese laboratories used ARTP mutagenesis to derive the erythritol overproducer non-GM mutant M53 strain from the wild-type SWJ-1b isolate [153]. Similarly, a group of research teams from Jiangnan University used mutagenesis combined with high-throughput screening to select an erythritol overproducer non-GM mutant strain from a wild-type isolate [326]. Interestingly, some genetic engineering technics were implemented in their project, but not for the design of the production strain: these tools were used to build a genetically-encoded erythritol biosensor strain (using an erythritol-responsive transcription factor to activate expression of an eGFP-encoding gene, leading to a fluorescence signal) that was used in the high-throughput screening test applied to the mutant strains generated through a combined UV/ARTP mutagenesis step [326]. The same authors pursued the optimization of their erythritol production bioprocess by applying an adaptative evolution strategy to the producing strain BBE-17 in order to enhance its thermotolerance during industrial fermentation, as evoked above in Section 4.5.3 [308]. Starting with a strain with an optimum temperature of 30 °C, they applied a progressive adaptive evolution scheme that allowed them to obtain, after 11 months of continuous cultivation and selection, an improved strain with an optimum temperature of 35 °C. However, the erythritol yield was considerably lower in this selected strain, which prompted the use of genetic engineering as a last resort: the authors performed a transcriptome analysis on their thermotolerant strain in order to identify the genes linked to this phenotype, reconstructed the thermotolerant phenotype using a surrogate Po1f strain and finally transferred the target gene modifications into a BBE-17Δ*Ku70*Δ*URA3* strain. This GM strain was able to produce a three-fold higher erythritol yield, without detrimental effects on cell growth, at a temperature of 33 °C compared to the parent BBE-17T strain [308]. Thus, such an example illustrates the limits of traditional mutagenesis, even when combined to adaptative evolution strategies, when used alone as an alternative to genetic engineering. The example of the serendipitous discovery of the *EUF1* gene, as described in Section 3.3.1, also illustrates the lack of reliability of some traditionally obtained mutations that could be much too easily prone to reversion [128].

It is to hope that a future easing of the regulation policy for the new GMOs, especially for gene edited/CRISPR-generated organisms, could allow the relieving of the regulatory constraints that presently limit their use in some of their numerous domains of application. Even though it would be difficult to determine what influence GMO regulations and societal acceptance could have had on the strategic choices of laboratories and companies, we can note that the major food-oriented applications of *Y. lipolytica* strains (citric acid, erythritol, KGA) have majorly favoured traditionally improved strains (cf. Section 2.3.1 and Section 3.3.3). If this tendency was to increase in the future, a more systematic exploration of the natural *Y. lipolytica* biodiversity for potential applications, leveraged by new mutagenesis technics (ARTP), adaptative evolution strategies and high-throughput screening technologies would constitute a valuable asset. Therefore, *Y. lipolytica* is in good position to become a biotechnological workhorse, through both traditional and genetic engineering pathways.

## Figures and Tables

**Table 1:** Selection of remarkable *Yarrowia lipolytica* wild-type or engineered (GM) strains of biotechnological interest deposited in public yeast collections (haploid, unless specified).

Strain Usual Name/Origin (Reference Number in Yeast Collections—cf. Table 2)	Genotype Phenotype	Remarkable Characteristics	Usual Applications
A-101/carwash effluents, Poland [123] not publicly available (a)	ND wild-type prototroph	robust growth on oil [123], high citric acid production [124], sequenced strain [125]	in situ soil bioremediation [51,52], citric acid production [124], UV-mutagenesis and metabolic engineering host for design of improved strains producing citrate or erythritol [70,126,127,128]
ACA-DC 50109 [aka LGAM S(7)1]/Greece [129] (b)	ND wild-type prototroph	very high lipid content and productivity [129,130], robust growth on crude glycerol, simultaneous high lipid and citric acid yields [131]	production of organic acids (notably citric acid) and SCO [129,130,131,132,133,134], including cocoa butter substitute [31,32], parent of evolved strain with increase oleaginicity [135], metabolic engineering host for design of improved GM strains [133,134]
ACA-DC 5033 [aka ACA-YC 5033]/acid sourdough, Greece [136]	ND wild-type prototroph	robust growth on crude glycerol, simultaneous high lipid and citric acid yields [24]	SCO, citric acid and polyol production [24,137]
ATCC 18942 [aka YB-423]/corn-processing plant, USA [138] (CBS 6124, CLIB 183, JCM 2320 and 8060, MUCL 29853, NBRC 1548, NRRL YB-423)	MatA MatB wild-type prototroph	diploid type strain [138], robust growth [139]	yeast biomass production [139]
ATCC 20362 [aka 2002]/USA	ND wild-type prototroph	robust growth, high lipid content and productivity [57]	degradation of petroleum crude oil (US patent 3856667A), metabolic engineering host for design of Dupont GM PUFA-producing platform (cf. Section 2.3.2) [57,58]
ATCC 48436/soil, Japan [140] (CBS 6303, CLIB 703, JCM 8054, NBRC 10073)	MatA wild-type prototroph	produces lipase activators [140]	lipase production [140], parent strain for Artechno highly lipolytic mutants used for bioremediation (cf. Section 2.3.1)
D 1805/France [141] (ATCC 20390)	MatA MatB wild-type prototroph	non-sporulating diploid, robust growth [141,142], self-cycling fermentation [143]	organic acid production [143]
H222/soil, Germany [10,144] (CLIB 80)	MatA wild-type prototroph	better fructose assimilation, high citric acid production [144], sequenced strain [34,37]	organic acid production, metabolic engineering host for design of improved GM strains [145,146]
NCIM 3589/marine waters, India [147]	ND wild-type prototroph	biofilm formation [148] emulsifier production [149]	gold nanoparticle production [102]
SWJ-1b/marine fish gut, China [4] (MCCC 2E00068)	ND wild-type prototroph	high level of crude protein [4]	citric acid and SCP production [150], metabolic engineering host for design of improved GM strains [151,152] ARTP-mutagenesis host for design of improved strains producing erythritol [153]
W29/sewage water *, France [11,154] (ATCC 20460, CBS 7504, CICC 1778, CLIB 89, NBRC 113670, NRLL Y-3178, VKPM Y-3178)	MatA wild-type prototroph	high secretion level of proteins [10,155], sequenced strain [36,156]	organic acid production [11], basis for the Po1 series of heterologous protein-producing GM strains and the JMY2566 GM strain for high-throughput applications (cf. Figure 2), basis for GM obese strains (cf. Section 3.1.4)
E129/GM from a W29 and ATCC 18942 crossing [10,157] (CLIB 121) (cf. Figure 2)	MatA, *lys11-23, leu2-270, ura3-302, xpr2-322* Lys^−^, Leu^−^, Ura^−^, Suc^+^, ΔAEP	able to grow on sucrose [10,15] deleted for alkaline extracellular protease [10]	heterologous protein production [155]
E150/GM from a W29 and ATCC 18942 crossing [10,157] (CLIB 122) (cf. Figure 2)	MatB, *his1, leu2-270, ura3-302, xpr2-322* His^−^, Leu^−^, Ura^−^, Suc^+^, ΔAEP	able to grow on sucrose [10,15] deleted for alkaline extracellular protease [10], reference sequenced strain [33,34]	reference for assembling and annotating genomes [33,34]
Po1d/GM from W29 [158] (CLIB 139) (cf. Figure 2)	MatA, *leu2-270*, *ura3-302*, *xpr2-322* Leu^−^, Ura^−^, Suc^+^, ΔAEP	able to grow on sucrose [15] deleted for alkaline extracellular protease [158]	heterologous protein production [19,20,158], metabolic engineering host for design of GM strains for multiple applications [19,20] metabolic engineering host for design of Oxyrane ERT-producing platform (cf. Section 2.3.2) [61]
Po1f/GM from W29 [159] (ATCC MYA-2613, CLIB 724, VKPM Y-3155) (cf. Figure 2)	MatA, *leu2-270*, *ura3-302*, *xpr2-322, axp1-2* Leu^−^, Ura^−^, Suc^+^, ΔAEP, ΔAXP	able to grow on sucrose [15] deleted for both extracellular proteases [159], sequenced strain [160,161]	heterologous protein production [19,20,159], metabolic engineering host for design of GM strains for multiple applications [19,20]
Po1g/GM from W29 [159] (CLIB 725) (cf. Figure 2)	MatA, *leu2-270*, *ura3-302::URA3, xpr2-322, axp1-2* Leu^−^, Suc^+^, ΔAEP, ΔAXP	able to grow on sucrose [15] deleted for both extracellular proteases, carry a pBR322 docking platform [159]	heterologous protein production [19,20,159], included in the YLEX kit for expression/secretion of heterologous proteins [19,20] (cf. Section 2.3.2)
Po1h/GM from W29 [18,43] (CLIB 882) (cf. Figure 2)	MatA, *ura3-302*, *xpr2-322, axp1-2* Ura^−^, Suc^+^, ΔAEP, ΔAXP	able to grow on sucrose [15] deleted for both extracellular proteases [18,43]	heterologous protein production [18,19,20,43], metabolic engineering host for design of GM strains for multiple applications [19,20]
Po1t/GM from W29 [18,43] (CLIB 883) (cf. Figure 2)	MatA, *leu2-270*, LEU2, *ura3-302::URA3*, *xpr2-322, axp1-2* Suc^+^, ΔAEP, ΔAXP	able to grow on sucrose [15] deleted for both extracellular proteases, carry a pBR322 docking platform, GM prototroph [18,43]	negative control for heterologous protein production by other Po1 strains [18,43]
JMY2566/GM from W29 [162] (CLIB 1779) (cf. Figure 2)	MatA, *leu2-270*, *ura3::pTEF-RedStar2-LEU2-zeta*, *xpr2-322* Ura^−^, ΔAEP, RedStar2	deleted for alkaline extracellular protease, fluorescent (red) strain, carry a zeta LTR sequences docking platform [162]	high-throughput mutant library screening [162]
VKM Y-2373/Russia (Fed) VKM Y-2412/Russia (Fed) not publicly available (c)	ND wild-type prototroph	natural overproducers of, respectively, (iso)citric acid [163,164] and KGA [165]	organic acid production [163,164,165], basis for traditionally obtained 704-UV4-A/NG50 mutant for improved (iso)citric acid production [163]
WSH-Z06/oil-polluted soil (refinery), China [166] not publicly available (d)	ND wild-type prototroph	thiamine-auxotrophic natural overproducer of KGA [166,167], sequenced strain [168]	KGA and keto acids production [167], basis for traditionally obtained hyper-producer mutants [168], metabolic engineering host for design of improved GM strains [169,170]
YB-392/gluten settler, USA YB-419/maize fiber tailings, USA (NRRL YB-392 and NRRL YB-419) YB-420, YB-566 and YB-567 (not publicly available, do not appear on online catalog)	ND wild-type prototrophs	biomass hydrolysate consumption, inhibitor tolerance, high lipid/fatty acid or sugar alcohol production (cf. Section 3.2) [118], sequenced strains [171]	five strains selected as promising candidates for industrial biocatalysis [118,171]

Strains are listed per alphabetic order of wild-type strain usual name, with their corresponding GM derivatives afterward (by chronological order of construction). (**a**) deposited at the collection of Wroclaw University of Environmental and Life Sciences. (**b**) deposited for safe at the Agricultural College of Athens-Dairy Collection under the name ACA-DC 5109, but not available from the collection: should be requested from the Laboratory of Food Microbiology and Biotechnology, Department of Food Science and Human Nutrition, Agricultural University of Athens. (**c**) deposited at VKM (cf. Table 2) but do not appear on online catalog. (**d**) deposited at the China Center for Type Culture Collection (CCTCC M207143). * Erroneously indicated as isolated from soil in other yeast collections. Abbreviations used, per order of occurrence in the table: GM, genetically modified; ND, non-determined or not disclosed; UV, ultraviolet light; SCO, single cell oil; PUFA, poly-unsaturated fatty acids; SCP, single cell protein; ARTP, atmospheric and room temperature plasma; ERT, enzyme replacement therapy; KGA, α-ketoglutaric acid.

**Table 2:** Main yeast culture collections proposing an important catalogue of publicly available strains of the *Yarrowia* genus or clade.

Country (Per Alphabetic Order)	Acronym of the Collection (WDCM Number)	Full Name of the Culture Collection	Website ISO Standard	Number of Strains of the *Yarrowia* Genus or Clade (*C*. for *Candida*)	Remarkable *Yarrowia lipolytica* Strains (Haploid, Unless Specified)
Belgium	BCCM/MUCL (WDCM 308)	Belgian Coordinated Collections of Microorganisms/MUCL Agro-food and Environmental Fungal Collection	http://bccm.belspo.be/about-us/bccm-mucl (accessed on 3 June 2021) ISO 9001:2015	29 *Y. lipolytica* + 3 *C. (Y.) alimentaria* 5 *Y. deformans* 1 *C. (Y.) galli* 1 *C. hispaniensis* 2 *C. (Y.) hollandica* 1 C. (Y.) osloensis * 4 *Y. yakushimensis* (including type strain for all) 2 *Yarrowia* sp.	MUCL 29853: diploid type strain (ATCC 18942)
China (PR)	CICC (WDCM 582)	China Center of Industrial Culture Collection	http://www.china-cicc.org (accessed on 3 June 2021) http://www.english.china-cicc.org (accessed on 3 June 2021) ISO 9001:2008 ISO 17025:2016; ISO 17034:2018	34 *Y. lipolytica* (with intended biotechnological applications indicated) 1 *Y. brassicae* (type strain)	W29 (CICC 1778) CICC 33063 for erythritol production CICC 31268 and 32291 edible and feed yeasts
	CGMCC (WDCM 550)	China General Microbiological Culture Collection Center	http://www.cgmcc.net (accessed on 3 June 2021) http://www.cgmcc.net/english/ (accessed on 3 June 2021) ISO 9001:2010; ISO 14001:2010	113 *Y. lipolytica*	
	MCCC (WDCM 1051)	Marine Culture Collection of China	http://www.mccc.org.cn/ (accessed on 3 June 2021) ISO 9001:2011	135 *Y. lipolytica* + 1 *Yarrowia* sp.	SWJ-1b (MCCC 2E00068): marine strain, for citric acid and SCP production
France	CIRM-Levures (WDCM 788)	Centre International de Ressources Microbiennes—Levures	https://www6.inrae.fr/cirm/Levures (accessed on 3 June 2021) https://www6.inrae.fr/cirm_eng/Yeasts (accessed on 3 June 2021) ISO 9001:2015	123 *Y. lipolytica* (including numerous GM laboratory strains) + 4 *Y. deformans* 1 C. *hispaniensis* (including type strain for both) 2 *Yarrowia* sp.	CLIB 183: diploid type strain (ATCC 18942) CLIB 703: type strain of *Candida paralipolytica*, for lipase production (ATCC 48436) E122 (CLIB 120): GM E129 (CLIB 121): GM E150 (CLIB 122): GM, sequenced (reference strain) H222 (CLIB 80): sequenced W29 (CLIB 89): sequenced W29 ura302 (CLIB 141): GM Po1a (CLIB 140): GM Po1d (CLIB 139): GM Po1e (CLIB 723): GM Po1f (CLIB 724): GM, sequenced Po1g (CLIB 725): GM Po1h (CLIB 882): GM Po1t (CLIB 883): GM
Germany	DSMZ (WDCM 274)	Leibniz-Institut DSMZ-Deutsche Sammlung von Mikroorganismen und Zellkulturen GmbH	http://www.dsmz.de/ (accessed on 3 June 2021) ISO 9001:2008 ISO 17025:2005; ISO Guide 34:2009	6 *Y. lipolytica* + 1 *Y. deformans*	
Greece	ACA-DC (WDCM 609)	Agricultural College of Athens-Dairy Collection	http://www.aca-dc.gr/ (accessed on 3 June 2021)	16 *Y. lipolytica* (isolated mainly from food)	ACA-DC 50109 ^§^, ACA-DC 5033: applied to citric acid and, respectively, SCO and polyol production
India	NCIM (WDCM 3)	National Collection of Industrial Microorganisms	https://www.ncl-india.org/files/NCIM/Default.aspx (accessed on 3 June 2021)	6 *Y. lipolytica* (erroneously indicated as *Y. lipolitica*)	NCIM 3589: marine strain, for gold nanoparticle production
Japan	NBRC (WDCM 825)	NITE (National Institute of Technology and Evaluation) Biological Resource Center	https://www.nite.go.jp/en/nbrc/index.html (accessed on 3 June 2021) https://www.nite.go.jp/nbrc/catalogue/NBRCDispSearchServlet?lang=en (accessed on 3 June 2021) ISO 9001:2008	20 *Y. lipolytica* + 1 *C. (Y.) phangngensis* 1 *Y. porcina* (type strain for both)	NBRC 1548: diploid type strain (ATCC 18942) NBRC 10073: type strain of *Candida paralipolytica*, for lipase production (ATCC 48436) W29 (NBRC 113670)
	JCM (WDCM 567)	Japan Collection of Microorganisms	https://jcm.brc.riken.jp/en/ (accessed on 3 June 2021) ISO 9001:2015	22 *Y. lipolytica* + 4 *Y. deformans* 1 *Y. keelungensis* 1 *Y. yakushimensis* (including type strain for all) 5 *Yarrowia* sp.	JCM 8057: type strain (ATCC 20177) JCM 2320 and 8060: diploid type strain (ATCC 18942) JCM 8054: type strain of *Candida paralipolytica*, for lipase production (ATCC 48436)
Netherlands	CBS-KNAW (WDCM 133)	CBS Filamentous fungi and Yeast Collection-Westerdijk Fungal Biodiversity Institute	http://www.westerdijkinstitute.nl/ (accessed on 3 June 2021) https://wi.knaw.nl/page/Collection (accessed on 3 June 2021) https://theyeasts.org/ (accessed on 3 June 2021) ISO 9001:2007	38 *Y. lipolytica* + 3 *C. (Y.) alimentaria* 1 *Y. brassicae* 1 *Y. bubula* 18 *Y. deformans* 1 *Y. divulgata* 2 *Y. galli* 2 C. *(Y.) hollandica* 1 *Y. keelungensis* 4 *Y. osloensis* 1 Y. parophoni * 1 *C. (Y.) phangngensis* 2 *Y. porcina* 4 *Y. yakushimensis* 2 C. *hispaniensis* (including type strain for all)	CBS 8108: type strain (ATCC 20177) CBS 6124: diploid type strain (ATCC 18942) CBS 6303: type strain of *Candida paralipolytica*, for lipase production (ATCC 48436) W29 (CBS 7504)
Russia (Fed)	VKPM (WDCM 588)	Russian National Collection of Industrial Microorganisms	https://vkpm.genetika.ru/ (accessed on 3 June 2021)	28 *Y. lipolytica*	W29 (Y-3178) Po1f (Y-3155): GM Po1f Ura^+^ (Y-3483): GM
	VKM (WDCM 342)	All-Russian Collection of Microorganisms	http://www.vkm.ru/Catalogue.htm (accessed on 3 June 2021)	17 *Y. lipolytica* + 1 *Y. deformans* (including type strain for all)	VKM Y-2373, VKM Y-2412: applied to organic acid production
USA	ATCC (WDCM 1)	American Type Culture Collection	http://www.atcc.org/ (accessed on 3 June 2021) ISO 9001:2015 ISO 13485:2016; ISO 17025:2017; ISO 17034:2016	132 *Y. lipolytica* + 1 *Y. deformans* 1 C. (Y.) phangngensis * (type strain for both)	ATCC 20177: type strain ATCC 18942: diploid type strain ATCC 48436: type strain of *Candida paralipolytica*, for lipase production D 1805 (ATCC 20390): non-sporulating diploid for organic acid production ATCC 20362: basis for Dupont PUFA-producing platform W29 (ATCC 20460) Po1f (ATCC MYA2613): GM
	NRRL (WDCM 97)	Agricultural Research Service (ARS) Culture Collection	http://nrrl.ncaur.usda.gov/ (accessed on 3 June 2021)	34 *Y. lipolytica* + 1 *C. (Y.) alimentaria* 1 *Y. bubula* 2 *Y. deformans* 1 *Y. divulgata* 1 *C. (Y.) galli* 1 C. *(Y.) hollandica* 1 *Y. keelungensis* 1 *C. (Y.) osloensis* 2 C. (Y.) phangngensis * 1 *Y. porcina* 1 *Y. yakushimensis* 2 C. *hispaniensis* (including type strain for all)	NRRL YB-423: diploid type strain (ATCC 18942) YB-392, YB-419, YB-420, YB-566 and YB-567: selected as promising candidate strains for industrial biocatalysis, all sequenced W29 (Y-63746)

Data compiled from WDCM (World Data Centre for Microorganisms—www.wfcc.info/ccinfo/home/ (accessed on 3 June 2021)) and from the collections’ websites. Accessed date for all websites: 3 June 2021. Abbreviations used, per order of occurrence in the table: SCP, single cell protein; GM, genetically modified; PUFA, poly-unsaturated fatty acids. *: indicated as their synonyms *oslonensis* on BCCM website, *parophonii* on CBS or *phangngaensis* on ATCC and NRRL websites. ^§^: deposited for safe but not available from the collection: should be requested from the Department of Food Science and Human Nutrition, Agricultural University of Athens.
